# Unraveling the molecular-pathological characteristics and cellular complexity of the tumor immune microenvironment in metastatic non-small cell lung cancer

**DOI:** 10.1186/s12964-025-02410-w

**Published:** 2025-09-29

**Authors:** Shiv Bharadwaj, Joanna Maria Mierzwicka, Lucie Vaňková, Petr Malý

**Affiliations:** Laboratory of Ligand Engineering, Institute of Biotechnology of the Czech Academy of Sciences, BIOCEV Research Center, Průmyslová 595, 252 50 Vestec, Czech Republic

**Keywords:** Genetic Alterations, Non-Small Cell Lung Cancer, Tumor Microenvironment, Cancer Stem Cells-, Metastasis

## Abstract

Metastatic non-small cell lung cancer (mNSCLC) cells carry heterogeneity, not only among different subtypes but also within a single tumor. Most evidence suggests that mNSCLC exploits specific molecular drivers and mechanisms to maintain physiology, metabolism, and immune evasion during tumorigenesis. Genome-wide association studies also revealed particular mutations in the oncogenic drivers supporting tumor cell proliferation and survival, resulting in aggressive and drug-resistant phenotypes of mNSCLC. While significant progress has been made in understanding mNSCLC at the genetic and molecular levels, a considerable gap remains in understanding the dynamic interplay between intrinsic factors—particularly key tumor-associated cells—and tumor immune microenvironment (TIME) during metastasis. Hence, this review highlights histological and genetic characteristics, emphasizes the clinical relevance of metastasis, and the roles of tumor-associated cells in shaping the immunosuppressive tumor microenvironment (TME) in mNSCLC. Understanding these intricate features and mechanisms is crucial for identifying novel therapeutic targets and improving strategies to combat mNSCLC progression in diagnosed patients.

**Graphical Abstract:**

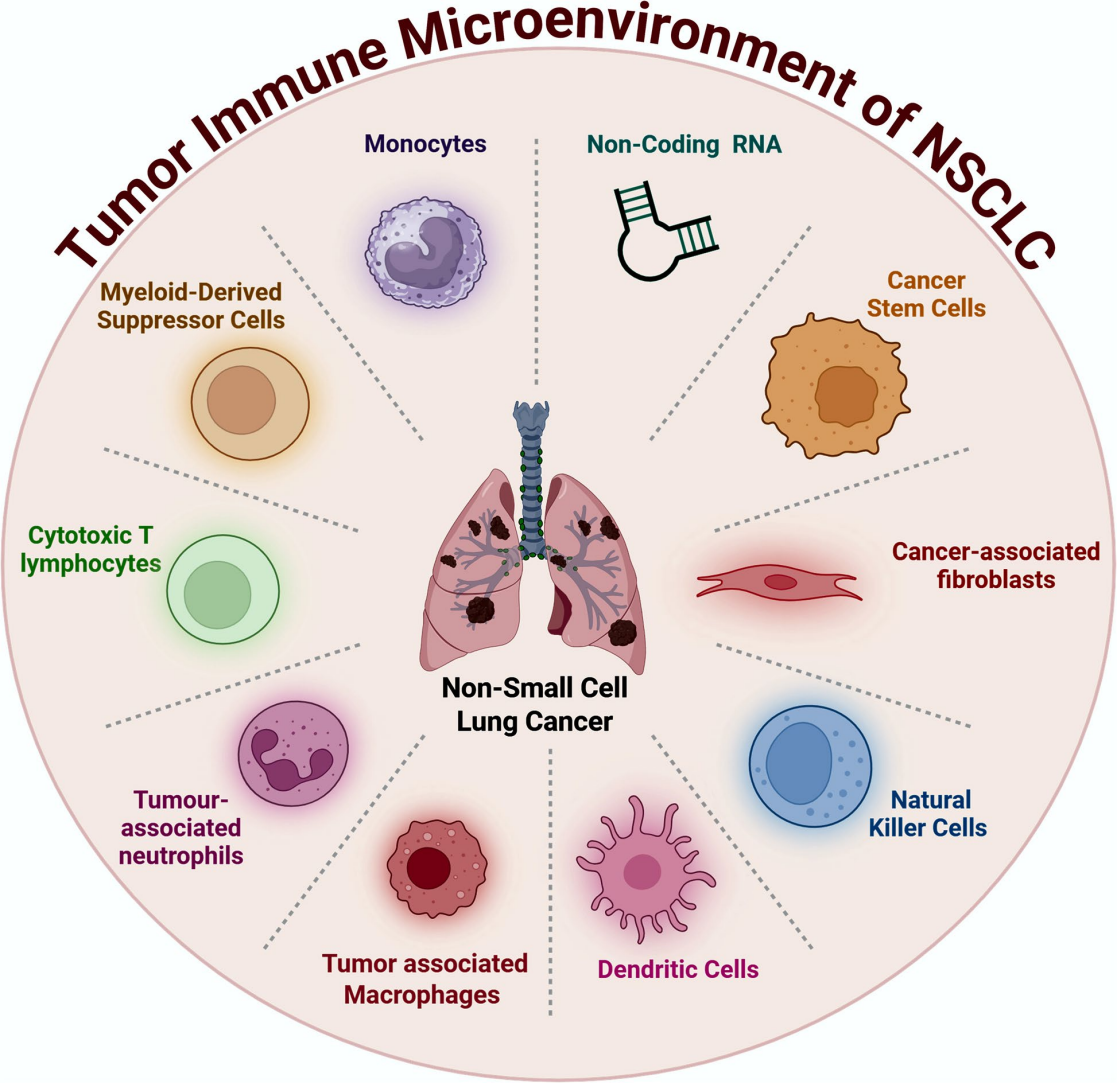

## Introduction

Despite modern innovations in diagnostic techniques and targeted therapies, lung cancer—characterized by frequent intratumoral heterogeneity [1, 2]—remains one of the deadliest cancers in the last two decades, contributing to high mortality rates in both men and women worldwide. As estimated by the International Agency on Cancer Research (IACR) of the World Health Organization (WHO), Lung cancer instigated approximately 9.96 million deaths worldwide in 2020, of which Lung cancer caused 1.8 million deaths, standing first in mortality [3]. Moreover, based on histological and molecular features, World Health Organization (WHO) has classified lung cancer into two major types: (i) non-small cell lung cancer (NSCLC), typically diagnosed in approximately 85% cases, and (ii) small cell lung cancer (SCLC), which covers the Outstanding 10%−15% of lung cancer patients [4]. A third less usual type of lung cancer is called large cell cancer (LCC), which accounts for approximately 10%−15% incidence of all NSCLC cases [5–7].

Despite recent advancements in cancer treatment—including surgery, radiation, chemotherapy, and targeted therapies—the prognosis for NSCLC remains poor as most of the lung cancer patients are diagnosed with locally advanced or metastatic disease [8]. Notably, metastatic NSCLC (mNSCLC) stands as a prime cause of treatment failure and low survival in diagnosed patients [9]. Consequently, prevention and inhibition of metastasis have been advocated as keys to NSCLC treatment [10]. Therefore, understanding the cellular and molecular biology of mNSCLC—including the complex interactions between tumor cells and their microenvironment—is of utmost significance in advancing cancer diagnosis and treatment.

Considering the recent literature available, in this review, we have comprehensively summarized the developments in the molecular-histologic characterization of mNSCLC with special emphasis on their clinical relevance in diagnosed lung cancer patients. Furthermore, we discuss the major non-genetic factors, including epithelial–mesenchymal transition (EMT) and non-coding RNAs (ncRNAs), as well as cellular complexity, including lung cancer stem cells (CSCs) and immune cell architecture, within the immunosuppressive tumor microenvironment (TME), which are primarily implicated in promoting or correlated with mNSCLC disease. Additionally, with the development of innovative platforms and ongoing clinical investigations in NSCLC, we have provided an outlook on the advanced strategies used to decipher the molecular complexity and clinical management of mNSCLC, along with their associated limitations.

## Dissection of key characteristics in NSCLC

**Fig. 1:**
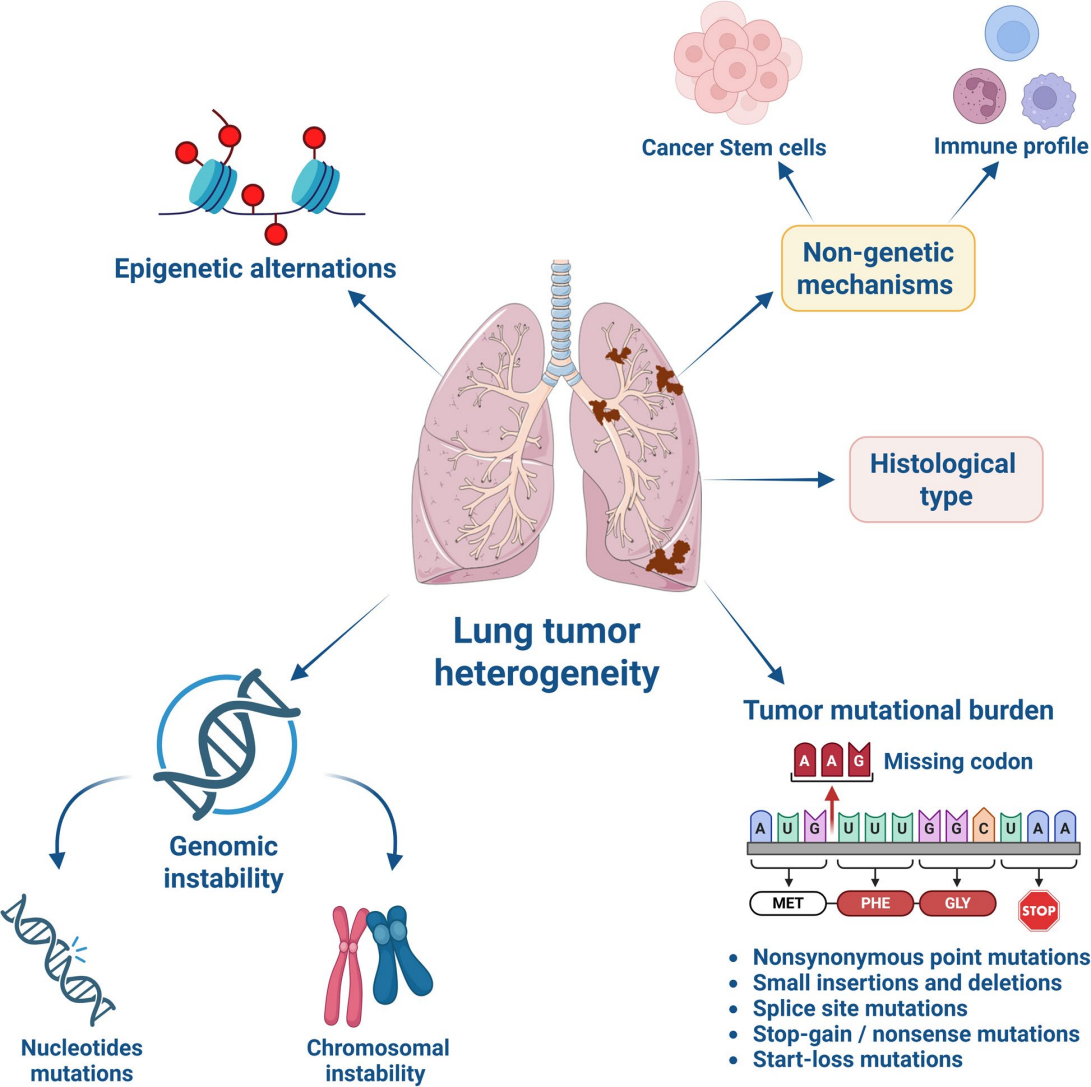
Schematic representation of multifactorial mechanisms, including genomic instability (nucleotide mutations and chromosomal instability), epigenetic alterations, non-genetic mechanisms (such as cancer stem cell and immune profile), histological type, and tumor mutational burden, driving heterogeneity in lung cancer. Created in BioRender. Bharadwaj, S. (2025) https://BioRender.com/tkhj3jq

In the current immuno-oncology era, histological subtyping remains an essential tool for predicting prognosis and guiding optimal therapy in lung cancer patients [11–13]. However, data analyses of mixed histologic subtypes—considering exposure history and shared genetic alterations—have demonstrated that the histological fate of cancer cells in most lung tumors is linked to transcriptomic features than genomic profiles [14]. This insight underscores the complexity of tumor biology, where both clinical and molecular heterogeneity in lung cancer, driven by genetic, non-genetic, and epigenetic mechanisms, continues to present major challenges for therapeutic selection (Fig. 1) [15–17]. In response, the Fundamental rules instituted by the 2011 International Association for the Study of Lung Cancer (IASLC)/American Thoracic Society (ATS)/European Respiratory Society (ERS) classifications laid the foundation for many advances in lung cancer therapy by promoting precise diagnosis through molecular and biomarker-based assessments, thereby encouraging the development of molecularly targeted immunotherapies [18]. Also, the 2021 WHO Classification of Thoracic Tumors again reaffirmed its 2015 recommendation for classification of the lung tumors primarily based on morphology, supported by immunohistochemistry, and subsequently confirmed through molecular techniques [18]. Furthermore, good clinical practice recommended guidelines in cancer treatment and details on how to grade Lung cancers using small biopsies are also briefed by the WHO Classification of Thoracic Tumors, 5^th^ edition, reviewed elsewhere [18]. Therefore, based on the available literature highlighting the prognostic significance of histology in NSCLC, this section summarizes the current knowledge on the major histological subtypes and associated genetic alterations in NSCLC, which are frequently implicated in mNSCLC.

### Histopathological classification

**Fig. 2:**
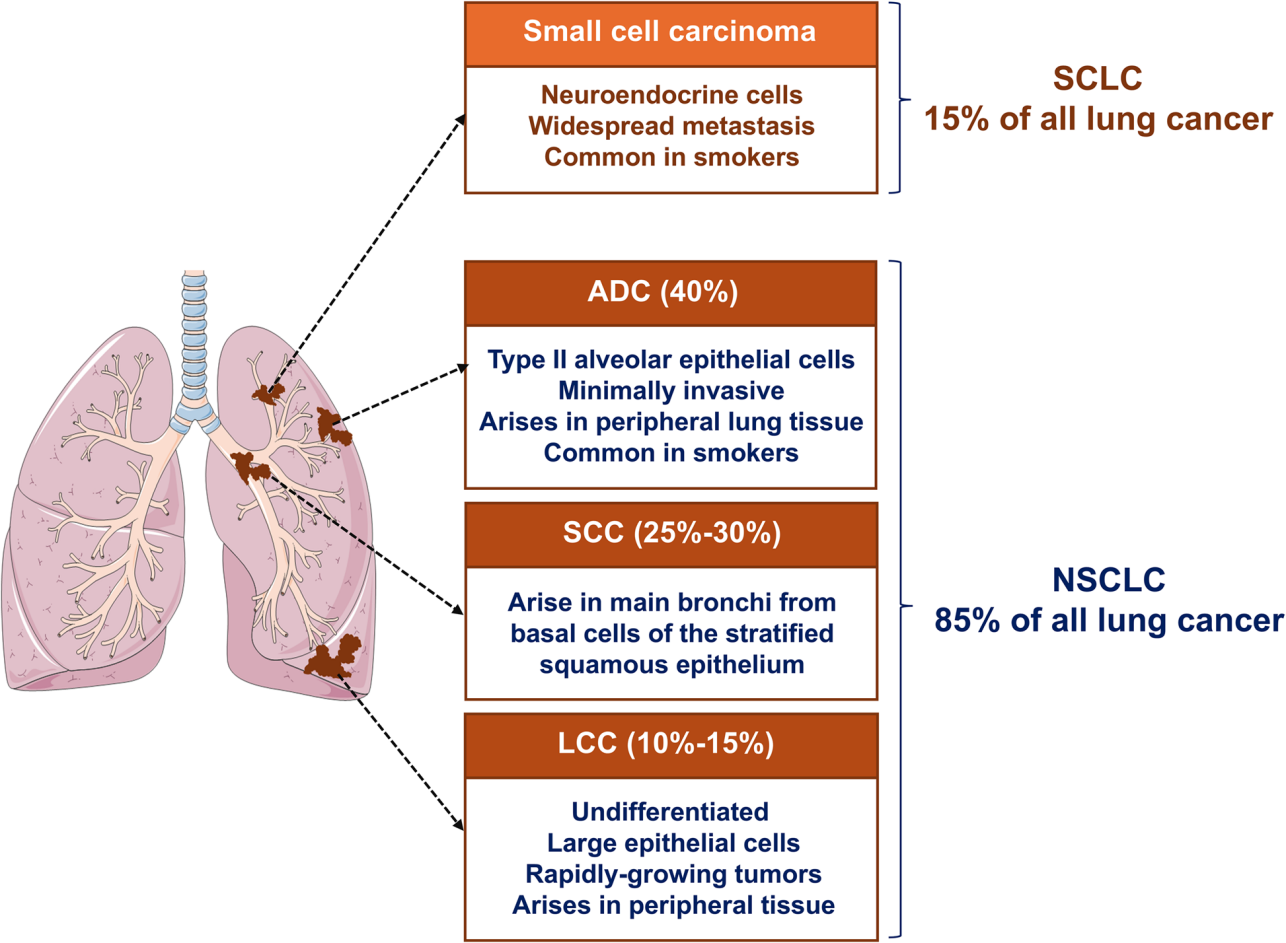
Histopathological and key attributes of lung cancer. The major histopathological types of lung cancer include small cell lung cancer (SCLC), which arises from neuroendocrine cells, and non-small cell lung cancer (NSCLC), which originates from epithelial cells. NSCLC is further classified into three main subtypes: adenocarcinoma (ADC), squamous cell carcinoma (SCC), and large cell carcinoma (LCC), as described in the published literature [26, 27]. Created in BioRender. Bharadwaj, S. (2025) https://BioRender.com/ejd767q

Histologically, NSCLC can be classified into adenocarcinomas (ADC, nearly 40%−60%) and squamous cell carcinoma (SCC, nearly 25%−30%) based on the site of origin [6, 7], as shown in Fig. 2. Typically, the ADC subtype originates from alveolar type II cells, while the SCC subtype commences from flat squamous cells of central airways in the lung [19, 20]. In terms of morphology, the ADC subtype is often characterized as a heterogeneous group of peripheral tumors in NSCLC patients [21–23]. Also, histological assessment of ADC shows papillary structures, gland formation, or solid growth with mucin production, while different variants have been reported within the ADC subtype. For example, bronchioalveolar carcinoma (BAC), a subtype of ADC, is associated with slower tumor growth, different clinical presentation, and a relatively better prognosis compared to other ADC subtypes [24]. On the other hand, SCC is commonly diagnosed in proximal bronchi and likely to remain localized. Also, histological characterization of the SCC subtype is typically direct, with reasonably wide keratinized regions and linked to inflammatory pathways, particularly in lesions lasting cavitation [25]. However, poorly differentiated types of SCC exhibit no keratinization and are commonly characterized as minor subsets of undifferentiated cells, which may recapitulate basal squamous epithelium layers. In this regard, indeed, basaloid variants of SCC have also been described, which showed substantially worse prognosis in comparison to conventional SCC [25]. Furthermore, other kinds of rare and poorly differentiated forms of the NSCLC subtypes, including large cell carcinoma (lung LCC), have been reported based on their histological features in NSCLC cases [6, 7].

As histological analysis is restricted to small biopsy samples, hence, absence of correct morphological features often averts a suitable LCC classification, which is particularly applicable in advanced stages of NSCLC and embraces high-grade undifferentiated primary or impracticable metastatic tumors. Nevertheless, the recent WHO classification has vividly reformed the standards for “anaplastic large cell carcinomas” by announcing a distinct group of “sarcomatoid carcinoma”, which contains only occasional tumor subtypes (clear cells, lymphoepithelial, and rhabdoid variants) [28]. A remarkable exclusion to the absence of distinguishing histologic measures is large cell neuroendocrine carcinoma (LCNEC), which was identified by Travis et al. [29] as a distinct tumor type with neuroendocrine characteristics while also exhibiting standard features of SCLC. Therefore, LCNEC has been suggested to be identified using neuroendocrine markers and recommended for exclusion in NSCLC studies due to its diverse biological and clinical characteristics. In addition to the three major NSCLC subtypes, a small NSCLC subset with mixed (sarcomatoid and adenosquamous carcinomas) or otherwise lacking specific histological and clinical features and/or indistinct from other subtypes is also reported in NSCLC patients [30]. Altogether, these histologic NSCLC subtypes are defined to possess distinct pathophysiology, clinical features, prognosis, and treatment sensitivity, as summarized in Table 1.

**Table 1:** 2015 WHO classification and main cellular characteristics of two major types of NSCLC based on cytologic samples and small biopsies in patients, adopted from Ref. [41]

Terminology	Morphological and staining features	Cellular characteristics
Adenocarcinoma morphologic patterns clearly present		Single cells or arranged in three-dimensional clusters; borders of cell clusters may be sharply defined
		Relatively abundant cytoplasm, typically more translucent than squamous cell carcinoma. Cytoplasm can range from distinctly homogeneous to granular to foamy because of the presence of vacuoles
		Single, eccentric, and round to oval with relatively smooth contours and minimal nuclear irregularity. Chromatin tends to be finely granular and evenly dispersed
Adenocarcinoma (list the patterns in the diagnosis)		Adenocarcinoma Predominant pattern:
		Lepidic
		Acinar
		Papillary
		Solid
		Micropapillary
Adenocarcinoma with lepidic pattern (if pure, list the differential diagnosis on the right and add a comment that an invasive component cannot be excluded)		Minimally invasive adenocarcinoma, adenocarcinoma in situ, or an invasive adenocarcinoma with a lepidic component
Invasive mucinous adenocarcinoma (list the patterns; use the term “mucinous adenocarcinoma with lepidic pattern” if pure lepidic pattern and mention the differential diagnosis listed on the right)		Invasive mucinous adenocarcinoma
		Minimally invasive adenocarcinoma or adenocarcinoma in situ, mucinous type
Adenocarcinoma with colloid features		Colloid adenocarcinoma
Adenocarcinoma with fetal features		Fetal adenocarcinoma
Adenocarcinoma with enteric features		Enteric adenocarcinoma
Nonsmall cell carcinoma, favor adenocarcinoma^b^	Morphological patterns are not present, but staining supports adenocarcinoma (i.e., TTF1 positive)	Adenocarcinoma (solid pattern may be just one component of the tumor)^b^
Squamous-cell carcinoma	Squamous cell morphologic patterns clearly present	Cells appear in cohesive aggregates, usually in flat sheets, and may appear as irregular shapes (e.g., spindle-shaped, and tadpole-shaped cells
		Abundant cytoplasm in irregularly shaped cells
		Central, irregular hyperchromatic nuclei, exhibiting one or more small nucleoli
Nonsmall cell carcinoma, favor squamous cell carcinoma^b^	Morphological patterns are not present, but staining supports squamous cell carcinoma (e.g., p40)	Squamous cell carcinoma (nonkeratinizing pattern may be a component of the tumor)^b^
Nonsmall cell carcinoma, NOS^a,c^	No clear adenocarcinoma or squamous morphologic patterns or staining pattern	Large cell carcinoma
	Squamous cell and adenocarcinoma morphologic patterns present (adenosquamous)	

Where,^a^metastatic carcinoma should be carefully excluded with a clinical and appropriate but judicious immunohistochemical examination; ^b^the categories do not always correspond to solid-predominant adenocarcinoma or non-keratinizing squamous cell carcinoma, respectivel;,and ^**c**^the non-small cell carcinoma-NOS pattern can be seen not only in large cell carcinomas but also when the solid, poorly differentiated component of adenocarcinomas or squamous cell carcinomas is sampled but does not express immunohistochemical markers or mucin

In general, as per the WHO recommendations, immunohistochemical (IHC) analysis of samples following morphological assessment should utilize thyroid transcription factor-1 (TTF-1 also known as NKX2-1) and keratin 7 (KRT7) as a marker for the ADC subtype while tumor protein 63 (p63) and ΔNp63 isoform of p63 (also written as Delta Np63, ΔNp63, or p40) as principal markers for the SCC subtype [31–33]. Although p63 has been shown to possess extremely high sensitivity for SCC [34], it has also been discovered to have low specificity due to its responsiveness in ADC, and in other tumor types, specifically for lymphomas [35]. Meanwhile, p40 is a comparatively new analytical marker for the SCC subtype, which showed equivalent sensitivity and superior specificity compared to p63 [36]. Besides, cytokeratin 5/6 has been suggested as a beneficial marker for SCC, remarkably in cases of undifferentiated or poorly differentiated NSCLC [37]. Importantly, it might be challenging to distinguish true primary SCC localized at distant metastatic sites solely based on IHC staining or microscopy analysis [38]. At the same time, occasional low expression of IHC markers can make it challenging to differentiate poorly differentiated ADC tumors from SCC [11, 23‒25]. Therefore, additional biomarkers have been identified that exhibited high potential in distinguishing the ADC and SCC subtypes of NSCLC, including melanophilin (MLPH), desmoglein 3 (DSG3), desmocollin 3 (DSC3), keratin 5 (KRT5), keratin 6 A (KRT6A), keratin 6B (KRT6B), surfactant-associated protein A2 (SFTA2), surfactant-associated protein A3 (SFTA3), calmodulin-like protein 3 (CALML3), and Transmembrane channel-like protein 5 (TMC5) [39, 40]. Overall, TTF-1 and p40 are among the most specific IHC markers, and in most cases are sufficient for distinguishing NSCLC subtypes.

### Genetic alterations

Despite gradual improvements in anticancer targeted therapy and immunotherapy, the efficacy of available treatments varies based on the mutational spectrum in tumor tissue [42]. Therefore, various molecular strategies have been used to evaluate multifocal NSCLCs, including array comparative genomic hybridization (CGH), loss of heterozygosity (LOH), broad genomic and functional screening assays for oncogenic alterations, and next-generation sequencing (NGS)-based genomic breakpoint analysis [43–49]. While the primary goal of molecular testing is to identify targetable genomic alterations, the integration of sequencing technologies into routine clinical practice has also enabled large-scale assessment of clonal relationships in NSCLCs. Consequently, multiple genetic alterations have been discovered in NSCLC, with a varied spectrum of mutations amongst ADC and SCC subtypes [50–52]. Interestingly, the frequency of identified mutations is typically ascribed to the total of homozygous deletions, somatic mutations, focal amplifications, and substantial up- or downregulation of gene expression profiles [8]. The most regular genetic modifications reported in the ADC and SCC subtypes are summarized in Table 2.

**Table 2:** Genetic subtypes and mutations linked to the histological types of NSCLC

Histological types	Genomic alterations	Genes		Frequency (%)	Ref
ADC	Fusions	*ALK*		3–7	[75]
		*ROS1*		2–3	[76]
		*RET*		1–2	[76]
		*NTRK1*		1–2	[62]
	Mutations	*EGFR*		30–40	[77]
		*BRAF*		0.5–5	[76]
		*KRAS*		20–30	[76]
		*MET*		3–4	[78]
		*PTEN*		1.7	[79]
		*PDGFRA*		6–7	[80]
		*PIK3CA*		5	[81]
		*TP53*		52	[82]
	Copy number gene alterations	Gains	*ERBB2*	2–5	[83]
			*EGFR*	10	[84]
			*MET*	2–5	[78]
			*TERT*	75	[85]
		Losses	*CDKN2A*	7	[86]
SCC	Fusions		*FGFRs*	23	[87]
	Mutations		*TP53*	79	[82]
			*NF1*	10	[80]
			*FGFR1*	20	[87]
			*FGFR2*	3	[69]
			*DDR2*	2–3	[88]
			*BRAF*	4–5	[89]
			*KRAS*	1–2	[90]
			*PDGFRA*	4	[80]
			*PIK3CA*	15	[81, 91]
			*PTEN*	10	[79]
	Copy number gene alterations	Gains	*Sox2*	65	[92]
			*PIK3CA*	15	[81, 91]
			*TP53*	79	[82]
		Losses	*CDKN2A*	15	[86]
			*PTEN*	8	[93]

Where, *ALK* Anaplastic Lymphoma Receptor Tyrosine Kinase, *ROS1* ROS Proto-Oncogene 1, Receptor Tyrosine Kinase, *RET* Ret Proto-Oncogene, *NTRK1* Neurotrophic Receptor Tyrosine Kinase 1, *EGFR* Epidermal Growth Factor Receptor, *BRAF* B-Raf Proto-Oncogene, Serine/Threonine Kinase, *KRAS* Kirsten Rat Sarcoma Viral Oncogene Homolog, *MET* MET Proto-Oncogene, Receptor Tyrosine Kinase, *PTEN* Phosphatase and Tensin Homolog, *PDGFRA* Platelet Derived Growth Factor Receptor Alpha, *PIK3CA* Phosphatidylinositol-4,5-Bisphosphate 3-Kinase Catalytic Subunit Alpha, *TP53* Tumor Protein P53, *ERBB2* Erb-B2 Receptor Tyrosine Kinase 2, *TERT* Telomerase Reverse Transcriptase, *FGFR1* Fibroblast Growth Factor Receptor 1, *FGFR2* Fibroblast Growth Factor Receptor 2, *FGFRs* Fibroblast Growth Factor Receptors (general), *NF1* Neurofibromin 1, *DDR2* Discoidin Domain Receptor Tyrosine Kinase 2, *Sox2* SRY-Box Transcription Factor 2,*CDKN2A* Cyclin Dependent Kinase Inhibitor 2A

In the lung ADC subtype, the most frequently mutated genes include the *Kirsten rat sarcoma viral oncogene homologue* (*KRAS*) and *epidermal growth factor receptor* (*EGFR*), as well as tumor suppressor genes such as *Kelch-like ECH-associated protein 1* (*KEAP1)*, *neurofibromin 1 (NF1), serine/threonine Kinase 11* (*STK11, or* also called l*iver kinase B1, LKB1)*, and *tumor protein p53* (*TP53)*. Notably, the majority of genetic mutations have been identified in exons 18–21 of the *EGFR* gene in the ADC subtype [53, 54]. To support this, for example, approximately 6%–12% of ADC cases have been found with concurrent mutations in *KRAS* and *LKB1*, and these co-mutant tumors caused a favorable metastatic phenotype, which was not noticed in other co-expressed mutations in ADC [55, 56]. Furthermore, recurrent amplifications in several potentially targetable oncogenes have since been identified in the ADC subtype, including *human epidermal growth factor receptor 2* (*HER2*), *fibroblast growth factor receptor 1* (*FGFR1*), and *mesenchymal-epithelial transition factor* (*c-MET*), along with fusion oncogenes such as *anaplastic lymphoma kinase* (*ALK)*, *neurotrophic tyrosine kinase receptor type 1* (*NTRK1*), *neuregulin 1* (*NRG1*), *rearranged during transfection* (*RET)*, and *c-ROS oncogene 1* (*ROS1*) [57–63]. For instance, chromosomal rearrangements involving the *RET* proto-oncogene have been identified in 1%–2% of ADC cases [64]. While *KIF5B* was initially recognized as the *RET* fusion partner, several alternative fusion partners have since been discovered, all of which are thought to promote ligand-independent dimerization and constitutive activation of *RET* [64]. Likewise, *ROS1* Translocations, cited to be 1%–2% of NSCLC cases [65, 66], are mostly found in patients who have never smoked, or who have a history of light smoking, and in tumors with ADC histology [66]. Collectively, these oncogenic modifications, among which some exhibit sensitivity to clinical inhibitors, are reported in the majority of ADC cases [50, 67, 68].

Moreover, as a relatively low whole-exome sequencing (WES) number has been reported for SCC compared to ADC subtype, possibly targetable mutations noted in ADC have no prevalence in the SCC subtype of NSCLC [69]. As a substitute, genes including *discoidin domain-containing receptor 2* (*DDR2*), *FGFR1-3*, and genes involved in the phosphoinositide 3-kinase (PI3K) pathway, were frequently appear to be regularly altered in the SCC subtype [69]. Subsequently, several of these mutants—except for those in the PI3K pathway—have been verified as driver mutations in preclinical studies for the SCC subtype [63, 70]. In general, commonly mutated genes in the SCC subtype include the tumor suppressors, *TP53* and *cyclin-dependent kinase inhibitor 2A* (*CDKN2A*), which translate to the p16^INK4a^ and p14^ARF^ proteins. Of note, *TP53* has been found mutated in approximately 90% of tumors while *CDKN2A* is deactivated in over 70% of SCC cases, primarily by epigenetic silencing via promoter methylation (21%), inactivating mutations (18%), exon 1β skipping (4%), or homozygous deletion (29%) [50, 69, 71]. Even though *EGFR* amplification mutation (*EGFR*^*amp*^) is also observed, unlike in the ADC subtype, actionable mutations in *receptor tyrosine kinases* (*RTKs*) are not often identified in the SCC subtype [50, 69, 71]. Likewise, *FGFR1*^*amp*^ has been predominantly described in the SCC subtype [63]. Also, SCC is characterized by harboring a higher frequency of chromosomal number alterations and substantially associated with an immunologically 'cold' tumor immune microenvironment (TIME) [72–74].

Furthermore, gene expression profiling suggests distinct subsets within each subtype that further assist in pathological classifications of NSCLC [94–99]. For example, the Cancer Genome Atlas (TCGA) conducted separate studies on ADC and SCC subtypes, aiming to provide a comprehensive molecular profile of each disease and uncover insights into the distinct molecular foundations of these NSCLC subtypes [99]. Interestingly, a significant proportion of TCGA lung cancer cases did not exhibit identifiable driver oncogenes while transcriptional and epigenetic profiling uncovered distinct disease subtypes, which are believed to reflect the downstream effects of oncogenic processes [99]. Besides, the National Comprehensive Cancer Network (NCCN) guidelines for NSCLC (2019, version 4) advocate testing of the tumor tissue to optimally identify mutations in *EGFR*, *KRAS*, *HER2*, *ALK*, *ROS1*, *MET*, *BRAF*, *RET,* and *NTRK* genes [100]. Additionally, NCCN revised guidelines (2021, version 2; the complete version of these guidelines is available at *NCCN.org*) again endorse broad panel molecular profiling (or testing using multiple limited assays) to optimally recognize actionable driver-gene alterations along with co-mutations and emerging biomarkers in patients with progressive NSCLC [101]. As a result, the identification of driver oncogenic alterations in a subset of tumors becomes pivotal to decipher the molecular differences in NSCLC, and thus, tumor genotyping is now considered a primary requirement for guiding treatment decisions in NSCLC patients [102]. Accordingly, the major molecular subtypes of the mNSCLC associated with dysregulated pathways and key characteristics have been identified and are condensed in Table 3.

**Table 3:** Summary of mNSCLC molecular subtypes, their frequent metastases, dysregulated pathways, and key characteristics

Molecular subtype	Metastases	Dysregulation or alternation	Characteristics	Ref
*ALK* positive NSCLC	Brain and liver	• Rearrangement (mostly translocations)	• Characterized by *EML4-ALK* fusion • Occurs in 4–7% of patients with NSCLC • High prevalence of PD-L1 expression (TPS of ≥ 1% in ∼50%) but are among the subtypes with the lowest TMB (median of < 3 mut/Mb) • Rearrangements induce autophosphorylation and constitutive activity of ALK and downstream signalling cascades such as PI3K and RAS • ADC with signet ring, or acinar histology	[60, 103–106]
*BRAF*-Mutant NSCLC	Brain, liver, and bones	• Class I V600 mutations associated with light or never smoking • Class II or III non-V600 mutations, which are mostly found in smokers	• Each accounting for ~ 50% of all *BRAF*-mutant NSCLCs • *BRAF*^V600E^ mutations are more frequent in women • TMB 3.5–4.9 mut/Mb for *BRAF*^V600^-mutant tumors and 8.8–9.6 mut/Mb for non-*BRAF*^V600^-mutant tumors; PD-L1 positivity in 73%–75% and 37%–56%, respectively • Activated by RAS and subsequently activating MEK and ERK (MAPK pathway), promotes ADC histological subtype	[107–112]
*EGFR*-positive NSCLC	Brain, bones, liver, and adrenal gland	• Overexpression • Activating EGFR mutations (exon 19 deletion and exon 21 L858 • *EGFR* exon 20 insertion (ex20ins) such as EGFR ex20ins FQEA (A763_Y764insFQEA)	• Overexpressed in 15–50% of NSCLC • Genetic alterations occur as exon 19 deletions (60%) or L858R missense substitutions (35%), both of which result in constitutive activation of the receptor leading to cell growth and proliferation • Ex20ins mutations vary from 1.8%–15% among EGFR mutations in NSCLC (e.g., L858R, exon 19 deletions) drive tumor progression and metastasis, and detected in 13–25% of ADC and SCC	[113–115]
*FGFR1*-poistive NSCLC	Brain	• Amplification	• *FGFR1* amplifications positively associated with *ALK* amplifications at the tissue level • FGFR1-ERK1/2-SOX2 axis promotes cell proliferation, epithelial–mesenchymal transition	[116–119]
*HER2*-Mutant NSCLC	Brain and bones	• Amplification • Overexpression • Mutation, e.g., primarily exon 20 insertions, Duplication or YVMA 776− 779 insertion (80 − 90%) or *HER2* rare point mutations: G660D, R678Q, E693K, and Q709L	• Amplification in 20% NSCLC • Overexpression in 6%–35% • Mutations in 1–2% • Low PD-L1 expression (TPS of < 1% in ~ 50%) and a low TMB (median of < 3 mut/Mb) • Activation of downstream PI3K/AKT signaling results in cellular proliferation, differentiation and migration	[120–125]
*KRAS*-Mutant NSCLC	Brain, lung-to-lung, and bone	• Point mutation at codons 12, 13, 14, and 60/61 • Most common at codon 12 (G12C)	• KRAS^G12C^, the most common genomic alteration in non-squamous NSCLC (up to 15% of all genomic alterations a high TMB (7.8 mut/Mb) and a high level of PD-L1 expression	[123, 126–129]
*MET*-Altered NSCLC	Brain, bones, and liver	• Overexpression • Amplification • Mutations • Alternative splicing, for example, METex14	• *MET* overexpression in 25–50% NSCLC cases • *MET* amplification in 7% NSCLC cases • *MET* exon 14 skipping mutations lead to decreased MET degradation, leading to high expression and increased activation • Enhances cell growth, differentiation, motility, and epithelial mesenchymal transition (EMT) in tumor cells through RAS/RAF/MAPK, PI3K/AKT/mTOR, WNT/β-catenin, and STAT pathways, and promotes cancer invasion	[120, 123, 130, 131]
*NTRK* fusion-positive NSCLC	Brain	• *Neurotrophin kinase* genes (*NTRK1, NTRK2,* and *NTRK3*) coding tropomyosin receptor tyrosine kinases (TRKA, TRKB, and TRKC) to form fusion protein with partner genes (e.g., rare *EML4::NTRK3* gene fusion)	• NTRK fusions are rare in NSCLC • Promoted activation of PI3K/AKT/mTOR, RAS/RAF/MAPK, and PLCγ pathways • Drives tumor growth and survival through a constitutively active fusion protein containing the TRK kinase domain	[123, 132–135]
*PD-L1*-high NSCLC	Multiple organs, including the brain, liver, and bones	• Overexpression	• At least 50% of tumor cells showed PD-L1 expression, while 34% of patients exhibited expression after relapse • Linked to immune evasion and aggressive metastasis	[136–138]
*PIK3CA*-Mutant NSCLC	Brain, bones, and lymph nodes	• Most common mutation is E545K and E542K on exon 9 and rare hotspot is kinase domain like H1047R on exon 20	• Frequency in lung SCC is high (11.4%) than in lung ADC (2.8%) • Leads to PI3K/AKT/mTOR pathway activation	[139–142]
*RET*-Rearranged NSCLC	Brain, liver, and bones	• Chromosomal rearrangements involve fusion partners such as KIF5B, CCDC6, NCOA4, and TRIM33 • 13 RET/PTC fusion proteins identified (RET/PTC1-PTC9)	• A low TMB (< 2.5 mut/Mb) but variable PD-L1 expression levels • Adenocarcinoma histological subtype • As frequent as 7–17% in NSCLC cases • Activation leads to RAS/RAF/MAPK, PI3K/AKT/mTOR and PLCγ signaling → cell proliferation, migration, and differentiation • Chimeric proteins constitutively dimerize, activating kinase domain leading to uncontrolled activation of MAPK and PI3K pathways	[123, 143–146]
*ROS1*-Rearranged NSCLC	Brain and liver	• Up to 24 fusion partners identified	• Occurs in 3% of NSCLC as rare but aggressive subtype • Adenocarcinoma histological subtype • ROS1 is a tyrosine kinase receptor with significant structural homology to ALK • Rearrangements/translocations give rise to fusions of functional ROS1 tyrosine kinase domain with other genes • Resulting constitutive activation drives transformation and activates SHP-1/SHP-2, JAK/STAT, PI3K/AKT/MTOR, and MAPK/ERK signaling leading to enhanced tumor cell survival and proliferation	[66, 123, 147–150]
*STK11/LKB1*-Mutant NSCLC	Lungs, liver, and brain	• Missense Mutations	• Occurs in 20–30% of NSCLC tumors *STK11* mutations are associated with an “immune cold” TME Often co-occurs with KRAS mutations and enhances tumor invasiveness	[151, 152]

In summary, depending on the NSCLC subtype, the genetic alterations vary, resulting in specific dysregulation of key pathways in mNSCLC, such as oxidative stress response, cell-cycle regulation, cell survival, apoptosis, and differentiation. Therefore, considering the distinct histological and genetic alterations, an enhanced understanding of the molecular mechanisms and subtypes driving mNSCLC progression may facilitate the expansion of targeted therapeutic approaches, potentially improving the overall survival (OS) of affected patients.

## Clinical relevance of metastatic NSCLC

**Fig. 3:**
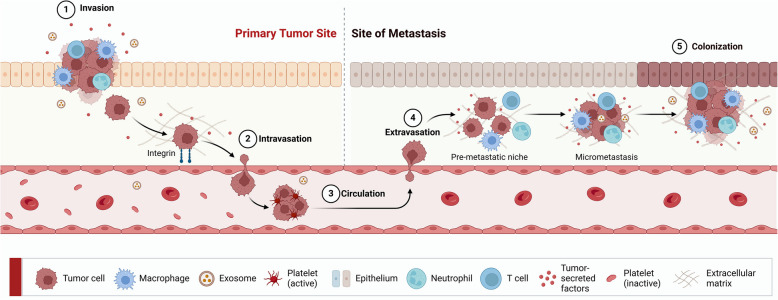
Illustration of multistep process involved in cancer metastasis. (1) Local invasion—cancer cells escape into surrounding tissues; (2) Intravasation—entry into the circulatory system, which is supported by interactions with endothelial cells and immune components; (3) Circulating tumor cells (CTCs)—CTCs survive in the circulatory system despite shear stress and immune attacks, and often form clusters; (4) Extravasation—CTCs adhere to endothelial cells or tissues at a distant site and exit the circulation. CTCs then invade the secondary site, forming a pre-metastatic niche and micrometastasis; and finally, (5) Colonization—disseminated tumor cells (DTCs) adapt to the new tissue microenvironment, evade immune surveillance, and establish a metastatic niche, leading to macrometastases. Created in BioRender. Bharadwaj, S. (2025) https://BioRender.com/6uvmhgn

Tumor metastasis, which refers to the systemic distribution and growth of tumor cells from the site of origin to distant body parts (tissue-destructive macro-metastases disrupt organ function), is a principal contributor to cancer-associated death [153–158], depicted in Fig. 3. In lung cancer, distant metastasis is defined as the dissemination of cancer cells from the primary sites to distant lymph nodes or distant organs, whereas lymph node metastasis (LNM) refers specifically to the spread of cancer cells to regional or sentinel lymph nodes. Characteristically, tumor cells undergo the epithelial-to-mesenchymal transition (EMT), which confers migratory, invasive ability, resistance, and stem-like characteristics [159, 160]. While metastasis is further orchestrated by intricate cell-to-cell signaling and interactions with the extracellular matrix (ECM) within the tumor microenvironment (TME), including cytokines (such as TGF-β, IL-6, and IL-8), stromal and immune cells, hypoxia, and ECM remodeling—collectively facilitating invasion, intravasation, immune evasion, and colonization at distant organs [161–166]. Moreover, the accumulation of DNA damage resulting in numerous genetic alterations, such as chromosomal translocations, deletions, or single-nucleotide polymorphisms (SNPs), has been acknowledged as a major contributor to tumorigenesis in lung cancer [167, 168]. Therefore, a small population of tumor cells can complete a treacherous journey involving survival in circulation, local invasion, intravasation, homing, extravasation into the parenchyma of distant tissues or organs, and adaptation to the new environment, as shown in Fig. 3. Ultimately, these cells lead to the outgrowth of secondary lesions, resulting in the clinical manifestation of metastatic disease [169, 170]. This section, therefore, aims to highlight the clinical relevance of mNSCLC by reviewing the current TNM staging system, metastatic patterns, and organotropism, as well as existing therapeutic strategies—all of which are essential for improving clinical outcomes and OS in patients with advanced NSCLC.

### TNM staging system and its revisions

In lung cancer, the TNM staging system—evaluating the size and characteristics of the primary tumor (T), the extent of lymph node involvement (N), and the presence or absence of metastasis (M)—is commonly employed to classify the disease's anatomical extent. This system provides a standardized framework for assessing disease severity, guiding treatment decisions, and predicting patient prognosis [171, 172]. The Union for International Cancer Control (UICC) and the American Joint Committee on Cancer (AJCC), with support from the International Association for the Study of Lung Cancer (IASLC) and the Staging and Prognostic Factors Committee (SPFC), regularly review and update the staging criteria [173]. Accordingly, in collaboration with the AJCC and IASLC, the UICC complied with and issued the recommendation for TNM staging in their 8th edition [174–176]. In this edition, specific combinations of TNM descriptors and lung cancer subtypes were classified into stages based on clinical studies [177, 178], are summarized in Table 4.

**Table 4:** TNM stage classification considering stage peculiarities and suggestive treatments for lung cancer [179, 180]

Tumor Stage	Tumor Staging			Characteristics	Treatment Suggestions
	**Size**	**Lymph Node**	**Metastasis**		
Occult carcinoma	TX	N0	M0	• Primary tumors cannot be assessed or proven by the presence of malignant cells in sputum or bronchial washings but not visualized with imaging or bronchoscopy • No regional lymph node metastasis • No distant metastasis	–
Stage 0	Tis	N0	M0	• Carcinoma in situ	
Stage IA1	T1a	N0	M0	• Tumor ≤ 3 cm in greatest dimension, surrounded by lung or visceral pleura, without bronchoscopic evidence of invasion more proximal than the lobar bronchus • Tumor ≤ 1 cm in greatest dimension	Surgical resection, Neoadjuvant PD1-PDL1 inhibition
Stage IA2	T1b	N0	M0	• Tumor ≤ 1 cm in greatest dimension	Surgical resection, Neoadjuvant PD1-PDL1 inhibition
Stage IA3	T1c	N0	M0	• Tumor > 2 cm but ≤ 3 cm in greatest dimension	Surgical resection, Neoadjuvant PD1-PDL1 inhibition
Stage IB	T2a	N0	M0	• Tumor with any of the following features: involvement of the main bronchus regardless of the distance from the carina; invasion of the visceral pleura; associated with partial or complete lung atelectasis or pneumonitis • Tumor > 3 cm but ≤ 4 cm in greatest dimension	Surgical resection, Neoadjuvant PD1-PDL1 inhibition
Stage IIA	T2b	N0	M0	• Tumor > 4 cm but ≤ 5 cm in greatest dimension	Surgical resection, Neoadjuvant PD1-PDL1 inhibition
Stage IIB	T1a-c T2a T2b T3	N1 N1 N1 N0	M0 M0 M0 M0	• Metastasis in ipsilateral peribronchial and/or ipsilateral hilar lymph nodes and intrapulmonary nodes, including involvement by direct extension • Tumor > 5 cm but ≤ 7 cm in greatest dimension or one that directly invades any of the following structures: parietal pleura, chest wall (including superior sulcus tumors), phrenic nerve, parietal pericardium; or separate tumor nodule or nodules in the same lobe	Surgical resection, Neoadjuvant PD1-PDL1 inhibition
Stage IIIA	T1a-c T2a-b T3 T4 T4	N2 N2 N1 N0 N1	M0 M0 M0 M0 M0	• Tumor measuring > 7 cm in greatest dimensions that invades any of the following structures: mediastinum, diaphragm, heart, great vessels, trachea, recurrent laryngeal nerve, esophagus, vertebral body, carina; or separate tumor nodules or nodules in a different lobe of the same lung	Chemotherapy followed by radiation or surgery, Neoadjuvant PD1-PDL1 inhibition
Stage IIIB	T1a-c T2a-b T3 T4	N3 N3 N2 N2	M0 M0 M0 M0	• Metastasis in contralateral mediastinal, contralateral hilar, ipsilateral or contralateral scalene, or supraclavicular lymph nodes • Metastasis in ipsilateral mediastinal and/or subcarinal lymph nodes	Combination of chemotherapy and radiation, Neoadjuvant PD1-PDL1 inhibition
Stage IIIC	T3 T4	N3 N3	M0 M0	• Metastasis in contralateral mediastinal, contralateral hilar, ipsilateral or contralateral scalene, or supraclavicular lymph nodes • Metastasis in ipsilateral mediastinal and/or subcarinal lymph nodes	Combination of chemotherapy and radiation, Neoadjuvant PD1-PDL1 inhibition
Stage IVA	Any T Any T	Any N Any N	M1a M1b	• Separate tumor nodules or nodules in the contralateral lung; malignant pleural effusion or pleural thickening or nodules or masses; malignant pericardial effusion or pericardial thickening or nodules or masses • Single distant (extrathoracic) metastasis in a single organ	Chemotherapy and palliative care with combination of either PD1-PDL1 or PD1-CTLA4
Stage IVB	Any T	Any N	M1c	• Multiple distant (extrathoracic) metastases in a single organ or multiple organs	Chemotherapy and palliative care with a combination of either PD1-PDL1 or PD1-CTLA4

Although the 8^th^ edition of the TNM classification established a correlation between stage classification and disease prognosis, there remains a substantial demand for a more precise prognostic model tailored to individual patients. For instance, the TNM-staging system based on the 8^th^ edition does not distinguish between unexpected pathological nodal metastasis in a single paratracheal node and the occurrence of enlarged nodes across multiple ipsilateral mediastinal stations [171]. Additionally, multiple analyses, even after controlling for potential confounders, have shown that M1c patients with a single metastatic lesion in an extrathoracic organ system have a distinct prognosis compared to those with metastases in multiple extrathoracic organ systems [181]. Therefore, in the 9^th^ edition of the TNM-staging system for lung cancer, the IASLC and SPFC recommend retaining the existing N0, N1, N2, and N3 descriptors, with the inclusion of new N2 subcategories: single-station involvement (N2a) and multiple-station involvement (N2b) [171]. Similarly, Fong et al. [181] validated and supported the retention of the 8^th^ edition M1a and M1b categories, while proposing a further refinement of the M1c category into M1c1 (involvement of a single extrathoracic organ system) and M1c2 (involvement of multiple extrathoracic organ systems). Accordingly, the recently issued 9^th^ edition of the TNM classification is in effect from January 2025 and incorporates these recommendations, including the revised N2 and M1c subcategories [182] (Table 5).

**Table 5:** TNM stage classification as recommended in the 9th edition of the TNM classification for lung cancer [182, 183]

T/M	Label	N0	N1	N2		N3
				**N2a**	**N2b**	
T1	T1a ≤ 1 cm	IA1	IIA	IIB	IIIA	IIIB
	T1ab > 1 to ≤ 2 cm	IA2	IIA	IIB	IIIA	IIIB
	T1c > 2 to ≤ 3 cm	IA3	IIA	IIB	IIIA	IIIB
T2	T2a Visceral pleura, central invasion	IB	IIB	IIIA	IIIB	IIIB
	T2a > 3 to ≤ 4 cm	IB	IIB	IIIA	IIIB	IIIB
	T2b > 4 to ≤ 5 cm	IIA	IIB	IIIA	IIIB	IIIB
T3	T3 > 5 to ≤ 7 cm	IIB	IIIA	IIIA	IIIB	IIIC
	T3 Invasion	IIB	IIIA	IIIA	IIIB	IIIC
	T3 Same lobe tumor nodules	IIB	IIIA	IIIA	IIIB	IIIC
T4	T4 > 7 cm	IIIA	IIIA	IIIB	IIIB	IIIC
	T4 invasion	IIIA	IIIA	IIIB	IIIB	IIIC
	T4 Ipsilateral tumor nodules	IIIA	IIIA	IIIB	IIIB	IIIC
M1	M1a Contralateral nodules	IVA	IVA	IVA	IVA	IVA
	M1a Pleural, pericardial effusion	IVA	IVA	IVA	IVA	IVA
	M1b Single extra-thoracic lesion	IVA	IVA	IVA	IVA	IVA
	M1c1 Multiple extra-thoracic lesions in a single organ system	IVB	IVB	IVB	IVB	IVB
	M1c2 Multiple extra-thoracic lesions in multiple organ system	IVB	IVB	IVB	IVB	IVB

### Metastatic patterns and organotropism

Metastatic patterns refer to the characteristic distribution and timing of cancer spread to distant organs. In NSCLC, even though advances in histological and molecular profiling have been made, approximately 20%–35% of cases are typically detected at stage III [184, 185], while nearly 30–40% present with distant metastasis at initial diagnosis [186, 187]. It is now well-established that metastatic dissemination is not random; instead, different cancers, including NSCLC, display a preference for certain organs, a phenomenon termed metastatic organotropism [188]. Consequently, approximately 50% of NSCLC cases are identified with metastases of the brain (47%), bone (36%), liver (22%), adrenal glands (15%), thoracic cavity (11%), and distant lymph nodes (10%) [9, 189, 190]. Additionally, limited studies have reported mNSCLC involvement in other organs at advanced stages [191–193]. Molecular insights suggested that the mechanism underlying organ-specific metastasis is multifactorial and supported by the development of pre-metastatic niches (PMN), aberrant immune cell accumulation, angiogenesis, and ECM remodeling [188, 194, 195]. Additionally, tumor-derived exosomes have been suggested to express unique integrins to facilitate the construction of a PMN with organ-specific resident cells to promote metastatic organotropism [196, 197]. Other reports have also discovered that systemic therapies can induce distinct alterations, predominantly in metastatic sites, which further supports the survival and progression of mNSCLC [198, 199]. For instance, approximately 60% of NSCLC cases develop metastases during cancer treatment [200–202]. Unfortunately, due to a lack of effective treatment modalities targeting advanced or distant mNSCLC, it results in a high mortality rate for lung cancer-related death [203].

Emerging data have shown that highly malignant mNSCLC cells acquire genetic alterations that allow them to grow and colonize distant organs [204–208]. In this direction, oncogenic driver mutations (also known as oncogene addiction), such as *KRAS*, *EGFR, ALK*, *ROS1*, *BRAF*, *HER-2*, *MET*, *NTRK*, and *RET*, mutations and inactivating mutations in tumor suppressor genes like *TP53*, *KEAP1*, *STK11*, and *NF1*, have been reported to promote mNSCLC disease [8, 209–212]. Moreover, as ADC is the most common histologic subtype of NSCLC, it has been significantly studied for organotropism compared to the SCC subtype [213–215]. In this context, a dedicated analysis performed by Lengel et al. [208] on ADC-specific organotropism through integration of clinicopathological features, temporal genomic relationships, and timing of site-specific metastatic spread discovered novel clinical insights, reported that younger, male patients with micropapillary or solid predominant tumors are highly susceptible to develop metastases, while histologic subtype showed no clear link to metastatic burden (number of metastatic sites) or organotropism. This study also identified genetic alterations, such as *SMARCA4* (*SWItch/Sucrose Nonfermentable, SWI/SNF related, Matrix associated, actin dependent regulator of chromatin, subfamily A, member 4*) and *TP53* mutations, linked to metastasis in primary ADC, noting that the risk associated with these alterations is disproportionately disseminated between organ sites [208]. Additionally, Lengel et al. [208] found that *SMARCA4* alterations were significantly correlated with a higher probability of bone-specific metastasis, whereas *CDKN2A* alterations were linked to a shorter time to bone metastasis [208]. Supporting this, other investigations also discovered potential association of both *SMARCA4* and *CDKN2A* alterations with poor outcomes in ADC-specific patients and significant correlation to organotropisms in mNSCLC [204, 216–219]. The MSK-MET cohort analysis, which involved prospective clinical sequencing of 25,000 patients, also revealed an association between adrenal gland metastasis and mutations in both *SMARCA4* and *TP53*, as well as a correlation of *TP53* mutations with CNS metastasis in NSCLC patients [220]. Likewise, Shih et al. demonstrated that genes involved in the Hippo pathway were amplified at a higher frequency in ADC-specific brain metastases [204], while Hippo pathway alterations were correlated with a shorter interval to CNS metastasis in ADC-specific patients [208]. Also, the frequency of brain metastases in *EGFR*-mutant NSCLC individuals was calculated to be as high as 70%, substantially higher compared to that in non-*EGFR*-driven NSCLC patients [221–223]. In another retrospective cohort study, the molecular features of *EGFR*-mutated NSCLC observed in brain metastases differed from those of primary tumors, suggesting that certain brain metastasis clones can also result in systemic progressions [224].

Although determining the relative molecular timing of metastatic spread is challenging in experimental models due to the possible dormancy of disseminated tumor cells, it can be inferred from sequencing human tumor samples [225]. In this context, data analysis from the TRACERx (TRAcking non-small cell lung Cancer Evolution through therapy (Rx); NCT01888601) [167], discovered that certain somatic alterations, like *MDM2* amplification in ADC and *TP53* mutations in the ADC and SCC subtypes, were practically always truncal, sustained during tumor evolution, occurring prior to metastatic divergence, and were connected with an amplified tendency of metastasis. In contrast, amplification of *Histone Cluster 1 H3 Family Member B* (*HIST1H3B*) in ADC was often absent or subclonal within the primary tumor, suggesting it may confer increased metastatic potential to a minority of cells or selective advantage in their new metastatic niche [226]. These findings suggest that evolutionary measures of tumor biology could assist in predicting metastatic outcomes and guide precision treatments targeting emerging metastasizing clones in the adjuvant setting. This report also highlight the importance of research autopsy programs, such as Posthumous Evaluation of Advanced Cancer Environment (PEACE; NCT03004755), which allows extensive sampling of metastases to accurately infer clonal relationships, patterns of dissemination, and inter- and intrametastatic heterogeneity, as well as emphasized on the critical need for dynamic and continuous temporal assessments of tumor evolution. Since obtaining multiple biopsies during a patient’s treatment course is often impractical, non-invasive methods like circulating tumor DNA (ctDNA) analysis have been recommended as vital tools to monitor the emergence of seeding clones and better understand disease progression [227, 228].

Overall, the potential clinical conjectures of tumor dispersion can be proficiently characterized by comparison of paired primary and metastatic tumors [167, 198, 208]. In concordance with these observations, metastatic organotropism (preferential spread to specific organs) and metastatic burden are shaped by molecular, histological and clinicopathological factors—including younger age, oncogenic driver mutations (such as *TP53* and *ERBB2* alterations), higher chromosomal instability, histological subtypes, and response to treatment [208, 214, 215, 229–231]—that influence the site, timing, and extent of metastatic spread. Therefore, advancing clinical understanding and the management of mNSCLC demands the integration of longitudinal genomic studies, a dynamic model of tumor evolution, non-invasive monitoring strategies, and the development of a unified theoretical framework to guide precision diagnosis, prevention, and treatment of metastatic disease.

### Therapeutic strategies for metastatic NSCLC

Clinical management of NSCLC varies based on specific histological, morphological, stage, and genetic features [232]. Advances in high-throughput methods, particularly NGS technology, have uncovered various oncogenic alterations in NSCLC, many of which have become targets for drug development [233]. Consequently, mNSCLC is broadly classified into oncogene-driven (e.g., *EGFR*, *ALK*, *ROS1*, *KRAS*, and *BRAF* alterations) and non-oncogene-driven subtypes, shaping drug development and regulatory strategies in patients [234]. This section reviews recent approved therapies and advancements in clinical trials for both oncogene-driven and non-oncogene-driven advanced NSCLC.

#### Oncogene-driven metastatic NSCLC

In oncogenic-driven metastatic NSCLC, ADC is the most common histologic subtype and frequently presents with high tumor mutation burden (TMB) and well-characterised, targetable oncogenic drivers [99, 235]. As a result, molecular testing for *EGFR*, *ALK*, *ROS1*, and *BRAF* mutations is advised for all newly identified advanced NSCLC patients [236] to guide targeted therapy selection [237, 238]. The most relevant phase III clinical trials and their major outcomes in oncogene-driven advanced NSCLC are summarized in Table 6**.**

**Table 6:** Summary of completed and ongoing randomized Phase III trials of targeted therapies for metastatic NSCLC with key oncogenic mutations (*EGFR*, *ALK*, *ROS1*, *KRAS*, *RET*, and *MET*)

Therapy (Target/Class)	Trial Name/ID	Design	Participants	Primary Endpoint(s)	Key Outcomes	Approval Status/Next Step
Alectinib (2nd-gen ALK TKI)	ALEX (NCT02075840)	Global, randomized, open‑label Phase III comparing alectinib 600 mg BID vs crizotinib 250 mg BID in treatment‑naïve ALK‑positive metastatic NSCLC	303 patients; no prior ALK-TKI or chemotherapy	PFS by BICR (RECIST v1.1)	• Median PFS: 25.7 vs 10.4 mo (HR 0.47; P < 0.001) • ORR: 82.9% vs 75.5% • CNS ORR: 59.7% vs 41.2% • Grade ≥ 3 AEs: 41% vs 50%	• FDA approved for first-line ALK-positive metastatic NSCLC (2017) • Ongoing real-world studies and comparisons vs later-gen inhibitors
	ALEX 5-Year Follow-Up (NCT02075840)	Long-term update of Phase III ALEX trial	Same 303 patients as ALEX	OS	• 5‑year OS rate: 62.5% vs 45.5% (HR ≈ 0.76) • Median OS: not reached vs 57.4 mo in crizotinib arm • Continued PFS benefit maintained	• Supports durable survival benefit; informs sequencing and real-world guideline updates (2020)
Osimertinib (3rd-gen EGFR TKI)	FLAURA (NCT02296125)	International, multicenter, randomized, double-blind comparing osimertinib vs gefitinib/erlotinib in first-line EGFR-mutant metastatic NSCLC	556 treatment-naïve EGFR-mutant metastatic NSCLC; no prior EGFR-TKI or chemotherapy	PFS by BICR (RECIST v1.1)	• Median PFS: 18.9 vs 10.2 mo (HR 0.46; P < 0.001) • ORR: 80% vs 76% • Median OS: 38.6 vs 31.8 mo	• FDA approved for 1L EGFR-mutant NSCLC (2018) • Global approvals late 2018
Osimertinib + Gefitinib (3rd-gen + 1st-gen EGFR TKIs)	FLAURA2 (NCT02841579)	Multicenter, randomized Phase Ib/III; addition of gefitinib to osimertinib after progression vs standard osimertinib continuation	~ 200 metastatic EGFR-mutant NSCLC progressing on first-line osimertinib; prior osimertinib only	OS and PFS by BICR	• Interim PFS improvement with combo vs continuation • Manageable safety profile	• Full data pending; may support new combination indication (2025)
Lorlatinib (3rd-gen ALK TKI)	CROWN (NCT03052608)	International, randomized, open-label Phase III comparing lorlatinib vs crizotinib in first-line ALK + metastatic NSCLC	296 treatment-naïve adults with ALK-positive metastatic NSCLC; no prior systemic/platinum or ALK-TKI therapy	PFS by BICR (RECIST v1.1)	• Median PFS: not reached vs 9.1 mo (HR 0.19) at ~ 60 mo median follow-up; 5-yr PFS: 60% vs 8% • Durable intracranial control (HR 0.06); median time to intracranial progression not reached • ORR higher across ALK variants; safety: Grade 3–4 AEs in 77% vs 57%; discontinuation 5% vs 6%	• FDA granted accelerated/regular approval (2019 US; global thereafter) based on earlier phase II/later phase III data • CROWN 5-year update confirms unprecedented PFS and intracranial efficacy • Ongoing analyses of OS and biomarker subgroups; informs guideline updates
	CodeBreaK 200 (NCT04303780)	Multicenter, randomized, open-label, active-controlled, 1:1 sotorasib (960 mg daily) vs docetaxel in previously treated, locally advanced/unresectable or metastatic NSCLC with KRAS p.G12C mutation	345 total (172 sotorasib; 173 docetaxel); ≥ 1 prior systemic therapy (largely platinum-based chemo)	PFS by BICR (RECIST v1.1)	• Median PFS: 5.6 mo vs 4.5 mo; HR 0.66; P = 0.0017 • 12-mo PFS rate: 24.8% vs 10.1% • ORR: 28.1% vs 13.2% • DCR: 83% vs 60% • Grade ≥ 3 TRAEs: 33.1% vs 40.4%	• FDA accelerated approval for KRAS G12C–mutated metastatic NSCLC after ≥ 1 systemic therapy (2021) • OS not improved (∼26% crossover) • Ongoing combo/earlier-line studies (CodeBreaK 101/300)
Adagrasib (Krazati) KRAS G12C	KRYSTAL-12 (NCT04685135)	Randomized, open-label, global phase III; adagrasib vs docetaxel in previously treated metastatic KRAS G12C NSCLC (2:1)	453 total (301 adagrasib; 152 docetaxel); all had prior platinum-based chemo + anti-PD-1/L1 therapy; crossover allowed	PFS by BICR (RECIST v1.1)	• Median PFS: 5.5 vs 3.8 mo (HR ≈0.58; P < 0.0001) • ORR: 32% vs 9% (P <.0001) • DCR: ~ 78% vs 50% • Duration of response: 8.3 vs 5.4 mo • Intracranial ORR: 24% vs 11% • Grade ≥ 3 TRAEs: ~ 47% vs 45.7%; discontinuations: 7.7% vs 14.3%	• FDA accelerated approval (US) based on KRYSTAL-1 (2022); conditional EU/UK approval KRYSTAL-12 confirmatory supports full approval (2024); OS data pending; ongoing combination trials (e.g. KRYSTAL-7)
Mobocertinib (TAK-788/AP32788; 3rd-gen EGFR TKI)	EXCLAIM-2 (NCT04129502)	Open-label, multicenter, randomized Phase III comparing mobocertinib 160 mg PO daily vs platinum-based chemotherapy (pemetrexed + cisplatin/carboplatin) as first-line treatment	318 patients with untreated locally advanced/metastatic NSCLC harboring EGFR exon 20 insertion mutations; no prior systemic therapy; crossover allowed on progression	PFS by BICR (RECIST v1.1)	• Median PFS: 9.6 vs 9.6 mo (HR 1.04; P = 0.803) • ORR: 32% vs 30% • Median DOR: 12.0 vs 8.4 mo • Median OS: not estimable vs 30.0 mo (HR 0.98) • Grade ≥ 3 AEs: diarrhea (20% vs 1%), anemia (6% vs 10%), lipase ↑ (6% vs 0%)	• Did not meet primary endpoint; efficacy not superior to chemotherapy • FDA and Takeda voluntarily withdrew mobocertinib (Exkivity) from EGFR ex20ins NSCLC indication (2023) • No subsequent Phase III follow-ups planned
Amivantamab + Lazertinib (EGFR/MET bispecific antibody + 3rd-gen EGFR TKI)	MARIPOSA (NCT04487080)	Global, randomized, open-label Phase III trial comparing amivantamab + lazertinib vs osimertinib in treatment-naïve metastatic NSCLC with EGFR exon 19 deletion or L858R mutation	1,074 patients with EGFR-mutant metastatic NSCLC; randomized 2:2:1 (combo vs osimertinib vs lazertinib)	PFS by BICR (RECIST v1.1)	• Median PFS: 23.7 vs 16.6 mo (HR 0.70; P < 0.001) • Intracranial PFS at 3 yrs: 38% vs 18% • OS: significant benefit over osimertinib • Grade ≥ 3 AEs manageable (e.g., rash, VTE)	• FDA approved for first-line EGFR-mutant metastatic NSCLC (2024) • EMA and other global approvals in 2025 • ongoing biomarker/resistance studies
Patritumab deruxtecan (HER3-directed ADC)	HERTHENA-Lung02 (NCT05338970)	Global, multicenter, open-label Phase III comparing patritumab deruxtecan (5.6 mg/kg Q3W) vs platinum-pemetrexed chemotherapy	586 patients with EGFR-mutant locally advanced or metastatic NSCLC after progression on a third-gen EGFR TKI (e.g., osimertinib); all had prior EGFR-TKI and ≥ 1 platinum-based regimen	PFS by BICR (RECIST v1.1)	• Met primary endpoint: significant PFS improvement vs chemo • No OS benefit vs chemo; OS target not met • Safety profile consistent with ADC class	• U.S. BLA withdrawn following OS results (2025) • Companies analyzing subgroups to identify potential responders; further regulatory discussions ongoing
Selpercatinib (RET inhibitor)	LIBRETTO-431 (NCT04194944)	Multicenter, randomized, open-label Phase III comparing selpercatinib vs platinum-based chemotherapy + pemetrexed ± pembrolizumab	261 treatment-naïve adults with RET fusion-positive, non-squamous metastatic NSCLC; no prior systemic therapy for metastatic disease; crossover allowed upon progression from control	PFS by BICR (RECIST v1.1) in ITT-pembrolizumab and ITT populations	• Met primary endpoint: HR ≈ 0.465 for PFS in ITT-pembrolizumab cohort • East Asian subgroup: median PFS not reached vs 11.1 mo (control); ORR 86.7% vs 61.0% • Intracranial activity, OS, ORR, DOR/DCR data consistent with benefit across subgroups; safety was manageable	• No approvals yet (as of mid-2025); selpercatinib currently approved in RET fusion-positive NSCLC based on earlier phase trials (LIBRETTO-001) • Full approval contingent on LIBRETTO-431 confirmatory data • Ongoing biomarker and QoL analyses including PRO outcomes
Capmatinib (MET inhibitor)	GEOMETRY Mono-1 (NCT02414139)	Multicohort, open-label Phase II study of capmatinib 400 mg BID in MET exon 14 skipping or MET-amplified metastatic NSCLC	160 patients with MET exon 14 skipping mutation: 60 treatment-naïve (1L), 100 previously treated with 1–2 lines (chiefly platinum-chemo)	ORR by BICR (RECIST v1.1); secondary: DOR, PFS, OS, safety	•Treatment-naïve: ORR 68% (95% CI 55–80%), median DOR 16.6 mo, median PFS 12.5 mo, median OS 21.4 mo. • Previously treated: ORR 44% (95% CI 34–54%), median DOR 9.7 mo, median PFS 5.5 mo, median OS 16.8 mo. Intracranial response in ~ 54% with brain mets; 98% disease control overall; grade 3–4 AEs in ~ 73% (peripheral edema, lipase ↑, ALT ↑); QoL maintained/improved across symptom scales	• FDA accelerated approval May 2020, converted to full approval August 2022, for MET exon 14 skipping mutation–positive metastatic NSCLC based on these results
Entrectinib (ROS1/TRK inhibitor)	NCT04603807	Ongoing randomized Phase III comparing entrectinib vs crizotinib in ROS1 fusion-positive metastatic NSCLC, including patients with CNS metastases	Untreated, ROS1-fusion NSCLC; no prior ROS1 TKI or systemic therapy; includes ≥ 30% with baseline CNS metastases	PFS in patients with baseline CNS metastases (primary); overall ITT PFS, OS, CNS-ORR secondary	Early phase data support intracranial efficacy and systemic ORR (~ 70% in single-arm), but Phase III data pending	• Repotrectinib has US approval (2023) based on single-arm data; entrectinib approved earlier via Phase II. • Full randomized trial results pending for labeling/regulatory update
Telisotuzumab vedotin (c-Met–directed ADC)	TeliMET NSCLC-01 (NCT04928846)	Global, randomized, open-label Phase III comparing telisotuzumab vedotin 1.9 mg/kg IV Q2W vs docetaxel 75 mg/m^2^ IV Q3W	600 adults with c-Met overexpressing, EGFR-wildtype, locally advanced/metastatic non-squamous NSCLC; ≤ 1 prior platinum-based chemotherapy line (adjuvant/neoadjuvant counts if progression ≤ 6 mo post-therapy); prior IO allowed	PFS by BICR (RECIST v1.1)	• Results pending (trial ongoing)	• FDA accelerated approval for previously treated advanced non-squamous NSCLC with strong (3 +) c-Met staining in ≥ 50% of tumor cells (as per VENTANA MET (SP44) RxDx Assay) (2025) • Full approval contingent on confirmatory results from TeliMET NSCLC-01

In the advanced-stage setting, recent clinical trials investigating specific oncogenic drivers, including *EGFR*, *ALK*, *ROS1*, *RET*, *ERBB2* (*HER2*), *BRAF, MET* exon 14 skipping (METex14), and *KRAS* alterations, have been reported with therapeutic efficiency have been reviewed elsewhere [239, 240]. For instance, the phase III AURA3 trial showed that third-generation tyrosine kinase inhibitor (TKI) osimertinib substantially enhanced progression-free survival (PFS) of platinum-based chemotherapy in advanced *EGFR*^T790M^-mutant NSCLC (10.1 vs. 4.4 months; p < 0.001) [241]. Whereas, combination strategies with third-generation EGFR TKIs (like osimertinib or lazertinib) and chemotherapy or amivantamab (a bispecific anti-EGFR/MET monoclonal antibody) [242, 243] demonstrated improved PFS versus monotherapy (FLAURA2: HR 0.62; MARIPOSA: HR 0.70), though overall survival data are pending [242, 243]. For *EGFR* exon 20 insertions positive NSCLC, first-line combination therapy with amivantamab plus chemotherapy also showed better response and PFS in comparison with chemotherapy alone; and this combination has been approved by the Food and Drug Administration (FDA)- and European Medicines Agency (EMA) for this population [244]. Similarly, TKIs targeting other druggable genomic alterations, such as *ALK* and *RET* fusions, reported improvement in outcomes in comparison with platinum chemotherapy [233, 245]. For example, the phase III LIBRETTO-431 trial demonstrated selpercatinib significantly increased PFS over standard care for RET-positive NSCLC (24.8 vs. 11.2 months; HR 0.46; *p* < 0.001) [245]. Meanwhile, lorlatinib, a third-generation ALK TKI, demonstrated a superior CNS activity and efficacy over crizotinib, and also benefits patients after second-generation ALK TKI failure [246–248]. Moreover, *KRAS*^G12C^, the most shared genomic alteration in ADC-specific NSCLC (accounting for up to 15% of all genomic alterations), has historically been difficult to target due to the protein’s essential function and structure; however, the discovery of the switch II pocket has enabled the development of specific inhibitors [249]. Consequently, the phase I/II trials of KRAS inhibitors sotorasib (AMG 510, Amgen) and adagrasib (MRTX849, Mirati Therapeutics) reported promising therapeutic efficacy [250, 251], but in phase III (KRYSTAL-12 and CODEBREAK-200 trials), they demonstrated only modest PFS benefits over docetaxel, with no overall survival gain for sotorasib; adagrasib OS data are pending in mNSCLC patients [252, 253]. Also, in the phase II KROCUS trial (NCT05756153), *KRAS*^G12C^ inhibitor fulzerasib in combination with cetuximab (chimeric monoclonal antibody targeting EGFR) as primary therapy showed an 80% objective response rate (ORR), 100% disease control rate (DCR), and activity in brain metastases, with manageable toxicity in mNSCLC patients [254]. Furthermore, immune checkpoint inhibitors (ICIs) were reported with limited efficacy in most oncogene-driven NSCLCs (except *KRAS*-mutated). For instance, meta-analysis conveyed poor monoimmunotherapy responses in *EGFR*-, *ALK*-, *HER2*-, *RET*-, and *ROS1*-altered positive NSCLC patients, but improved outcomes with combination immunotherapy [255]. Additionally, *KRAS*^G12C^, *BRAF*^non−V600E^, and *MET*^*amp*^ alterations may respond better to ICIs, warranting further assessment [255].

Patients with NSCLC harboring oncogenic alterations and treated with TKIs often develop resistance, leading to disease recurrence. Key oncogenic driver mutations—including *EGFR* (Exon 19 deletion, Exon 20 insertion, G719X, S768I, L858R, L861Q), *ALK*, *ROS1*, *RET* fusions, *MET* exon 14 skipping (METex14), *BRAF*^V600E^, *KRAS*^G12C^, *NTRK*, *HER2*, and *NRG1*—along with their corresponding targeted therapies and resistance mechanisms, have been recently reviewed [207, 233, 256–260]. In this setting, few effective treatment options remain; for example, antibody–drug conjugates (ADCs) are emerging as targeted options for oncogene-driven NSCLC. ADCs are divided into biomarker-selected (e.g., HER2 and MET overexpression) and biomarker-agnostic types (e.g., TROP2, and HER3), while several antibody drug conjugates are being tested in the specific context of oncogene-addicted NSCLC [261]. For instance, TROP2-targeting ADC, sacituzumab govitecan (IMMU-132)—an antibody–drug conjugate targeting TROP2 (trophoblast cell-surface antigen 2) antigen and delivering the cytotoxic agent SN-38, has shown promising anticancer activity and practicable safety in patients with advanced NSCLC formerly treated with chemotherapy and immunotherapy [262]. Also, Telisotuzumab vedotin (Teliso-V), c‑Met protein-targeted ADC, is being evaluated in MET-overexpressing and EGFR-mutant resistant NSCLC [263, 264]. At present, trastuzumab–deruxtecan is the only ADC approved for the treatment of *HER2*-mutant NSCLC [265, 266].

Overall, rapid and comprehensive molecular profiling is essential to identify actionable oncogenic drivers that guide treatment in both perioperative and metastatic NSCLC. While adjuvant osimertinib and alectinib are now the standard-of-care (SOC) for resected early-stage *EGFR*- and *ALK*-positive NSCLC, respectively, targeted therapies are under investigation in clinical trials to expand their use in the perioperative setting, aiming to replicate their success in mNSCLC. Besides, clinical trials are essential to assess the utility of perioperative setting for less common alterations (< 5% prevalence), including *HER2*, *MET*, *ROS1, RET*, *BRAF*, and *NTRK* alterations*,* and Brain metastases are common in these subtypes (10–50% incidence), and future studies should refine trial endpoints, treatment durations, and combinatorial strategies [267, 268]. Therefore, imminent studies should consider these mutated NSCLC subgroups and refine trial endpoints, treatment durations, and combinatorial strategies.

#### Non-oncogene-driven metastatic NSCLC

Historically, the standard first-line treatment for mNSCLC was a platinum-doublet chemotherapy regimen with or without pemetrexed (a folate antimetabolite) maintenance therapy, depending on tumor histology (pemetrexed being reserved only for non-squamous histology) [269]. However, the treatment landscape for non-oncogene-driven mNSCLC has evolved dramatically with the advent of ICIs targeting programmed cell death protein 1 (PD-1) and its ligand PD-L1. These agents have demonstrated superior survival outcomes compared to the previous second-line SOC, docetaxel, across four phase III randomized controlled trials (RCTs) [270–273], and have produced durable responses in a subset of patients [269, 274, 275]. These trials established PD-L1 expression of  ≥ 50% as a key biomarker predicting response to immune checkpoint blockade (ICB) monotherapy activity [276]. As a result, subsequent trials investigated ICB monotherapy in the first-line setting, showing substantial improvements in PFS and overall survival (OS) compared with platinum-based chemotherapy [136, 277, 278]. These results led to regulatory approval of first-line ICB monotherapy for NSCLC patients with PDL1 ≥ 50% by the EMA, while the FDA also approved pembrolizumab for patients with PD-L1 ≥ 1%. To date, except for cytotoxic T-lymphocyte–associated protein 4 (CTLA-4) inhibitors in a selective set of patients, the addition of other immunomodulatory agents—such as T cell immunoreceptor with Ig and ITIM domains (TIGIT) blockade, multikinase inhibitors, and TGF-β inhibitors—to first-line ICB monotherapy has not yielded significant improvements in NSCLC patients [279–282]. A summary of phase III clinical trials in advanced NSCLC without oncogenic driver mutations is provided in Table 7.

**Table 7:** Phase III trials in non-oncogenic metastatic NSCLC (driver mutation negative) including immuno-oncology, combination therapies, and ADCs

Therapy (Target/Class)	Trial Name/ID	Design	Participants	Primary Endpoint(s)	Key Outcomes	Approval Status/Next Step
Pembrolizumab (anti–PD‑1)	KEYNOTE-024 (NCT02142738)	Randomized, global multicenter, open‑label, 1:1 pembrolizumab vs platinum‑based Chemotherapy in 1L PD‑L1 ≥ 50% NSCLC	305 total (154 pembro; 151 chemo)	PFS; OS (secondary)	• PFS: 10.3 mo (95% CI 6.7–NR) vs 6.0 mo (95% CI 4.2–6.2); HR 0.50 (95% CI 0.37–0.68); P < 0.001 • OS (5-yr FU): 26.3 mo (95% CI 18.3–40.4) vs 13.4 mo (95% CI 9.4–18.3); HR 0.62 (95% CI 0.48–0.81) • 5-yr OS rate: 31.9% vs 16.3% • ORR: 44.8% vs 27.8%	FDA approved 1L pembro for PD‑L1 ≥ 50% metastatic NSCLC (2016)
Pembrolizumab (anti–PD-1)	KEYNOTE-042 (NCT02220894)	Randomized, global multicenter, open-label 2:1 pembrolizumab 200 mg q3w vs platinum-based Chemotherapy in 1L PD-L1 TPS ≥ 1%	1,274 treatment-naive metastatic NSCLC patients (TPS ≥ 1%)	OS	• TPS ≥ 50%: OS 20.0 mo vs 12.2 mo; HR 0.69 (95% CI 0.56–0.85); P = 0.0003 • TPS ≥ 20%: OS 17.7 mo vs 13.0 mo; HR 0.77 (95% CI 0.64–0.92); P = 0.0020 • TPS ≥ 1%: OS 16.7 mo vs 12.1 mo; HR 0.81 (95% CI 0.71–0.93); P = 0.0018	Approved for 1L PD-L1 TPS ≥ 1% NSCLC (2018)
Pembrolizumab + pemetrexed + platinum (anti–PD-1 + chemotherapy)	KEYNOTE-189 (NCT02578680)	Randomized, global multicenter, double-blind, 2:1 pembrolizumab vs placebo each with pemetrexed + carboplatin/cisplatin for 4 cycles, then pembrolizumab/placebo + maintenance pemetrexed until progression or toxicity	616 patients with previously untreated metastatic non-squamous NSCLC without EGFR/ALK alterations (410 pembro; 206 placebo)	OS, PFS	• Median PFS: 8.8 mo vs 4.9 mo (HR 0.52; P < 0.001) • 12-mo OS rate: 69.2% vs 49.4% (HR 0.49; P < 0.001) • 5-yr OS rate: 19.4% vs 11.3% (HR 0.60; 95% CI 0.50–0.72) • 5-yr PFS rate: 7.5% vs 0.6%	FDA approved 1L nonsquamous NSCLC (2018)
Pembrolizumab + carboplatin + paclitaxel/ nab-paclitaxel (anti–PD-1 + chemotherapy)	KEYNOTE-407 (NCT02775435	Randomized, global multicenter, placebo-controlled, 1:1 pembrolizumab vs placebo + chemo (4 cycles), then Maintenance up to 35 cycles; crossover allowed	559 treatment-naive metastatic squamous NSCLC patients	OS; PFS	• Median OS: 17.2 mo vs 11.6 mo; HR 0.71 (95% CI 0.59–0.85) • Median PFS: 8.0 mo vs 5.1 mo; HR 0.62 (95% CI 0.52–0.74) • 5-yr OS rate: 18.4% vs 9.7% (HR 0.71) • 5-yr PFS rate: ~ 10.8% vs ~ 3.5% • ORR: ~ 62.2% vs ~ 38.8% in primary analysis	FDA approved for 1L metastatic squamous NSCLC regardless of PD‑L1 (2018)
Pembrolizumab + Ipilimumab (anti–PD‑1 + anti-CTLA‑4) vs Pembrolizumab monotherapy	KEYNOTE‑598 (NCT03302234)	Randomized, global multicenter, double-blind 1:1 pembrolizumab + ipilimumab vs pembrolizumab + placebo in 1L PD-L1 TPS ≥ 50% metastatic NSCLC (no EGFR/ALK)	400 treatment‑naive metastatic NSCLC patients with PD‑L1 TPS ≥ 50%	OS; PFS	• Stopped for futility: interim analysis showed no incremental OS or PFS benefit with the combination over pembrolizumab alone • Toxicity: higher grade 3–5 TRAEs and serious AEs with combo (e.g., ≥ CTCAE 3 immune-mediated events)	Trial stopped early for futility; increased toxicity in combo arm; pembrolizumab monotherapy remains standard of care
Atezolizumab (anti–PD‑L1)	IMpower110 (NCT02409342)	Randomized, global multicenter, open‑label 1:1 atezolizumab vs Platinum Agent (Cisplatin or Carboplatin) + (Pemetrexed or Gemcitabine)	572 total; EGFR/ALK wild-type; 205 with high PD-L1 expression (TC ≥ 50% or IC ≥ 10%)	OS (hierarchical by PD‑L1); PFS	• High PD-L1 subgroup (205 pts): median OS 20.2 mo vs 13.1 mo (HR 0.59; P = 0.01) • Overall ITT: OS and PFS hierarchically favored atezo in EGFR/ALK WT and high bTMB subgroups (details not released) • Safety (all pts): any-grade AEs 90.2% vs 94.7%; grade 3–4 AEs 30.1% vs 52.5%	Approved 2020 for 1L PD-L1 high metastatic NSCLC (2020)
Atezolizumab + nab-paclitaxel + carboplatin (anti–PD-L1 + chemotherapy)	IMpower130 (NCT02367781)	Randomized, global multicenter, open-label 2:1 atezolizumab combo vs carboplatin + nab-paclitaxel for 4 cycles, then maintenance	724 chemo-naive stage IV non-squamous NSCLC patients without EGFR/ALK alterations	OS; PFS	• Median OS (ITT-WT): 18.6 mo vs 13.9 mo; HR 0.79 (95% CI 0.64–0.98); p = 0.033 • Median PFS (ITT-WT): 7.0 mo vs 5.5 mo; HR 0.64 (95% CI 0.54–0.77); p < 0.0001 • Grade ≥ 3 TRAEs: neutropenia 32% vs 28%; anemia 29% vs 20% • Serious AEs: 24% vs 13%; treatment related deaths 2% vs < 1%	Approved for 1L metastatic non squamous NSCLC without EGFR/ALK aberrations (2019)
Atezolizumab + carboplatin + nab-paclitaxel (anti–PD-L1 + chemotherapy)	IMpower131 (NCT02367794)	Randomized, global multicenter, open-label 1:1:1 A + CP vs A + CnP vs CnP for 4–6 cycles; maintenance atezolizumab for A-arms until progression	1,021 chemo-naive patients with stage IV squamous NSCLC (EGFR/ALK WT)	PFS; OS	• PFS (A + CnP vs CnP): 6.3 mo vs 5.6 mo; HR 0.71 (95% CI 0.60–0.85); P = 0.0001 • OS (A + CnP vs CnP): 14.2 mo vs 13.5 mo; HR 0.88 (95% CI 0.73–1.05); P = 0.16 (not statistically significant) • OS PD‑L1‑high subgroup: HR 0.48 (95% CI 0.29–0.81) • Safety (A + CnP vs CnP): Grade 3–4 TRAEs 68.0% vs 57.5%; serious AEs 47.9% vs 28.7%	Did not achieve OS benefit; no subsequent squamous‑NSCLC approval
Atezolizumab + Platinum + Pemetrexed	IMpower132 (NCT02657434)	Randomized, global multicenter, open‑label 1:1 atezolizumab + chemo (APP) vs chemo alone (PP)	578 patients with untreated metastatic nonsquamous NSCLC, EGFR/ALK WT	PFS; OS	• Median PFS: 7.6 mo vs 5.2 mo; HR 0.60; P < 0.0001 < br > • Median OS: 17.5 mo vs 13.6 mo (HR 0.86; NS) at final 2019 analysis	Approved as SOC in some regions; global approval pending
Atezolizumab + Bevacizumab + Carboplatin + Paclitaxel (anti–PD-L1 + anti-VEGF + chemotherapy)	IMpower150 (NCT02366143)	Randomized, global multicenter, open-label 1:1 ABCP vs BCP	1,202 chemo-naive metastatic nonsquamous NSCLC patients (EGFR/ALK WT)	OS, PFS	• ACP vs BCP (cutoff Sep 13, 2019): median OS 19.0 vs 14.7 mo; HR 0.84 (95% CI 0.71–1.00); not statistically significant • ABCP vs BCP (39.8 mo follow-up): median OS 19.5 vs 14.7 mo; HR 0.80 (95% CI 0.67–0.95); sustained OS benefit • PD-L1 subgroups (SP142/SP263): longer OS with both ACP and ABCP vs BCP in PD-L1-high/positive; similar OS in PD-L1-negative • Safety consistent with earlier reports	FDA approved 1L nonsquamous NSCLC (2018)
Nivolumab + Ipilimumab + 2-cycle chemotherapy (anti-PD-1 + anti-CTLA-4 + chemotherapy)	CheckMate-9LA (NCT03215706)	Randomized, global multicenter, open-label 1:1 NIVO + IPI + 2 cycles platinum-doublet vs 4 cycles platinum-doublet chemotherapy	719 untreated metastatic NSCLC patients (360 NIVO + IPI + C; 359 C alone)	OS	• Median OS: 18.9 mo vs 12.9 mo; HR 0.67 (95% CI 0.46–0.97) • 2-yr OS: 45% vs 32% • Median PFS: 6.8 mo vs 5.0 mo; HR 0.66 • ORR: 38% vs 25%	Approved (USA/EU/Japan) For metastatic or recurrent NSCLC (2020)
	CheckMate 9LA 6-Year Update	Post-hoc long-term analysis of the Phase III trial	All originally randomized patients (n = 719)	OS at 6 years	• HR for death: 0.74 (95% CI 0.63–0.87) • 6-yr OS: 16% vs 10% • Durable benefit across PD-L1 (< 1%/≥ 1%) and histology (squamous/non-sq.) subgroups	Endorsed by guidelines (ESMO/ASCO/NCCN) for 1L chemo-immunotherapy in NSCLC (2025)
Nivolumab (anti–PD-1)	CheckMate 017 (NCT01642004)	Randomized, global multicenter, multicenter, open‑label 1:1 Nivolumab 3 mg/kg q2w vs docetaxel 75 mg/m^2^ q3w	272 patients with advanced squamous NSCLC after prior chemotherapy	PFS:	• Median OS: 9.2 mo (nivo) vs 6.0 mo (docetaxel); HR 0.59; P < 0.001 • 1-yr OS: 42% vs 24% • ORR: 20% vs 9% • Fewer grade 3–4 TRAEs	FDA Approved for 2L + squamous NSCLC (2015)
Nivolumab (anti–PD-1)	CheckMate 057 (NCT01673867)	Randomized, open-label Nivolumab 3 mg/kg q2w vs docetaxel 75 mg/m^2^ q3w	582 patients with advanced non-squamous NSCLC after prior chemo	OS	• Median OS: 12.2 mo (nivo) vs 9.4 mo (docetaxel); HR 0.73; P = 0.0015 • ORR: 19% vs 12% • Fewer grade 3–4 TRAEs (10% vs 54%)	FDA approved in 2L nonsquamous NSCLC (2015)
	CheckMate 017 + 057 (5-year follow-up)	Pooled long-term follow-up of both trials	854 (combined	OS		Long-term benefit confirmed (2021)
Nivolumab + Ipilimumab (anti–PD-1 + anti–CTLA-4)	CheckMate 227 Part 1a (NCT02477826)	Randomized, global multicenter, open-label 1:1:1 nivolumab + ipilimumab vs nivolumab alone vs chemotherapy (PD-L1 ≥ 1%); and nivolumab + ipilimumab vs nivolumab + chemo vs chemo (PD-L1 < 1%)	1,739 treatment-naive metastatic/recurrent NSCLC patients (EGFR/ALK wild-type), stratified by PD-L1 status	OS in PD-L1 ≥ 1%; PFS in high TMB cohort	• 5-yr OS PD-L1 ≥ 1%: 24% (NIVO + IPI) vs 14% (chemo); HR not specified • 5-yr OS PD-L1 < 1%: 19% vs 7 • Median Duration of response: 24.5 mo vs 6.7 mo (≥ 1%); 19.4 mo vs 4.8 mo (< 1%) • Long-term benefit regardless of PD-L1; 39% 5-yr OS when combining ≥ 1% and < 1% cohorts • Safety: no new safety signals; good QoL in survivors	Supported 1L approval of nivolumab + ipilimumab in PD-L1 ≥ 1% metastatic NSCLC (2020)
Tremelimumab + Durvalumab + Chemotherapy (anti–CTLA-4 + anti–PD-L1 + chemotherapy)	POSEIDON/(NCT03164616)	Randomized, global multicenter, open-label 1:1:1 T + D + CT vs D + CT vs CT alone for 4 cycles; maintenance per arm	1,186 treatment-naive metastatic NSCLC patients (EGFR/ALK wild-type; PD-L1 evaluable)	PFS; OS	• Triplet vs CT (OS): HR 0.77 (95% CI 0.65–0.92); median OS 14.0 mo vs 11.7 mo; P = 0.0030 • Triplet vs CT (PFS): HR 0.72 (95% CI 0.60–0.86); median PFS 6.2 mo vs 4.8 mo; P = 0.0003 • D + CT vs CT (OS): trend HR 0.86 (95% CI 0.72–1.02); median OS 13.3 mo vs 11.7 mo; P = 0.0758 • TMB: exploratory benefit in TMB‑high subgroup, but testing not required due to limited incremental gain • Safety: Grade 3–4 TRAEs 51.8% (T + D + CT) vs 44.6% (D + CT) vs 44.4% (CT); imAEs higher with triplet but mostly low grade and manageable	FDA approved T + D + CT for 1L metastatic NSCLC (no EGFR/ALK); D + CT is FDA‑authorized via the same label expansion (2022)
Sugemalimab (anti–PD-L1)	GEMSTONE-302 (NCT03789604)	Randomized, global multicenter, double-blind, multicenter trial comparing sugemalimab plus Chemotherapy vs placebo plus Chemotherapy in 1L metastatic NSCLC	479 treatment-naive patients with stage IV squamous or non-squamous NSCLC, no EGFR/ALK/ROS1/RET alterations	PFS; OS	• Median PFS: 8.3 months vs 5.6 months; HR 0.50 (95% CI: 0.39–0.62); P < 0.0001 • Median OS: 21.2 months vs 15.3 months; HR 0.68 (95% CI: 0.54–0.85); P = 0.0004 • ORR: 45.3% vs 29.5%; P = 0.0002 • Safety: Grade ≥ 3 TRAEs 18.3% vs 39.9%; no new safety signals	Approved in China, UK, and EU for first-line metastatic NSCLC without EGFR/ALK/ROS1/RET alterations (2020)
Cemiplimab (anti–PD 1)	EMPOWER-Lung 1 (NCT03088540)	Randomized, global multicenter, open‑label 1:1 cemiplimab vs platinum‑based Chemotherapy in 1L PD‑L1 ≥ 50% metastatic NSCLC	~ 712 patients with metastatic squamous or non-squamous NSCLC, PD-L1 ≥ 50%, EGFR/ALK/ROS1 WT	OS; PFS; ORR	• Median OS: 26.1 mo vs 13.3 mo; HR 0.57 (95% CI 0.46–0.71); P < 0.0001 • Median PFS: 8.1 mo vs 5.3 mo; HR 0.51 (95% CI 0.42–0.62); P < 0.0001 • ORR: ~ 46.5% vs ~ 20.6%; durable responses—median duration ~ 16–17 mo; PFS rate ~ 20.8% at 3 yrs • 5-yr OS rate: ~ 29% vs 15% • Safety: Grade 3–4 TRAEs 18.3% vs 39.9%; consistent safety profile, no new signals	FDA approved for 1L metastatic NSCLC with PD‑L1 ≥ 50% (2021)
Datopotamab deruxtecan (Dato-DXd)	TROPION-Lung01 (NCT04656652)	Randomized, global multicenter, open‑label	604 patients (299 Dato‑DXd; 305 docetaxel) with previously treated advanced or mNSCLC with or without actionable genomic alterations	PFS, OS, ORR	• Median PFS: 4.4 mo (95% CI 4.2–5.6) vs 3.7 mo (95% CI 2.9–4.2); HR 0.75 (95% CI 0.62–0.91); P =.004 • Median OS: 12.9 mo (95% CI 11.0–13.9) vs 11.8 mo (95% CI 10.1–12.8); HR 0.94 (95% CI 0.78–1.14); P =.530 • Nonsquamous subgroup: PFS 5.5 vs 3.6 mo (HR 0.63 [0.51–0.79]); OS 14.6 vs 12.3 mo (HR 0.84 [0.68–1.05]) • Squamous subgroup: PFS 2.8 vs 3.9 mo (HR 1.41 [0.95–2.08]); OS 7.6 vs 9.4 mo (HR 1.32 [0.91–1.92]) • Safety: Grade ≥ 3 TRAEs 25.6% vs 42.1%; any-grade ILD/pneumonitis 8.8% vs 4.1%	FDA Priority Review; EU marketing application withdrawn by AstraZeneca
Telisotuzumab vedotin (c‑Met‑directed ADC)	TeliMET NSCLC‑01 (NCT04928846)	Recruiting; multinational multicenter, randomized, open‑label vs investigator’s choice (confirmatory)	Previously treated adults with locally advanced/metastatic, EGFR‑wildtype, nonsquamous NSCLC and high c‑Met overexpression	PFS, OS	–	Trial ongoing; efficacy and safety data pending

To broaden the applicability of ICIs—specifically anti-PD1 and anti-PD-L1 agents across a larger patient population, several combination strategies have been evaluated. These include dual ICI regimens (anti-PD-1 or anti-PD-L1 combined with an anti-CTLA-4 such as ipilimumab or tremelimumab), with or without a short course of chemotherapy, as well as combinations of anti-PD-1/PD-L1 agents with chemotherapy [283–285]. These investigations have demonstrated that dual ICI combinations, with or without chemotherapy, can improve PFS and OS across different NSCLC histologies, although the benefit appears less pronounced in PD-L1–negative tumors. The optimal immunomodulatory strategy, however, remains an area of ongoing investigation [283–285]. Notably, the phase III KEYNOTE-189 trial reported that pembrolizumab combined with pemetrexed and platinum-based chemotherapy substantially increased PFS and OS in previously untreated patients with metastatic non-squamous NSCLC lacking *EGFR* or *ALK* mutations [11].

Additionally, ADCs have been investigated as a novel treatment strategy for non-oncogene-driven NSCLC, though their benefits have been limited beyond HER2-driven tumors [265]. For example, datopotamab deruxtecan, a TROP2-directed ADC, demonstrated a modest improvement in PFS compared to docetaxel in the second-line setting (4.4 vs. 3.7 months; HR 0.75; 95% CI, 0.62–0.91; *P* = 0.004), but did not show an OS benefit (HR 0.90; 95% CI, 0.72–1.13), and was associated with a 25% incidence of grade ≥ 3 adverse events [286]. Similarly, sacituzumab govitecan—another TROP2-directed ADC with a topoisomerase inhibitor payload (SN-38)—failed to improve overall OS compared to docetaxel as second-line treatment in the EVOKE-1 trial (median OS 11.1 vs. 9.8 months; HR 0.84; 95% CI, 0.68–1.04; *P* = 0.534), although it suggested potential benefit in patients previously refractory to ICI treatment (HR 0.75; 95% CI, 0.58–0.97) [287].

Furthermore, for patients with poor performance status (ECOG ≥ 2) who are ineligible for platinum-doublet chemotherapy, the phase III IPSOS trial demonstrated that single-agent ICIs, such as atezolizumab, provide superior outcomes and improved tolerability compared with single-agent chemotherapy [288]. However, treatment options after progression on both ICIs and chemotherapy remain limited. Docetaxel—either as monotherapy or in combination with an antiangiogenic agent—remains the SOC, though it provides only modest improvements in PFS and OS for most patients [269]. To date, phase III RCTs investigating new combination strategies have not reported meaningful improvements in the overall patient population [289–291], although some patients may receive substantial benefit [292]. These findings highlight the urgent need for biomarker-based clinical trials to refine and optimize post-ICI treatment strategies in mNSCLC patients.

## Non-genetic factors promoting metastatic NSCLC

The immune system's homeostasis and the dynamic processes occurring within the TME are overly complex, posing a considerable challenge to fully understand the functional interactions between immune cells and tumors. Additionally, accurately characterizing the phenotypes of tumor-associated immune cells and their responses following anticancer treatment remains a significant challenge [293, 294]. However, mounting clinical evidence suggests that the TME status in NSCLC patients can serve as prognostic indicators after anticancer treatment. Given the TME’s pivotal role in both metastasis and clinical decision-making, the following sections will discuss the clinically investigated major intrinsic non-genetic factors supporting mNSCLC within the TME.

### Epithelial-to-mesenchymal transition

**Fig. 4:**
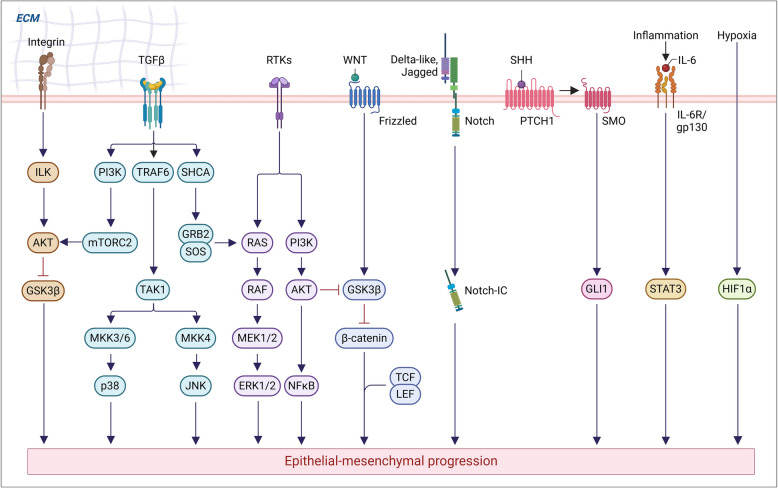
Key signaling pathways driving epithelial-mesenchymal transition (EMT) in NSCLC, including integrin-ECM interactions, transforming growth factor beta (TGF-β) signaling, receptor tyrosine kinases (RTKs), Wnt, Notch, Hedgehog (SHH), inflammatory cytokines (IL-6), and hypoxia-induced responses. All these pathways converge to drive EMT, enhancing cancer cell invasiveness, metastasis, and therapy resistance, adopted from ref. [296, 297]. Created in BioRender. Bharadwaj, S. (2025) https://BioRender.com/3zji3ge

Epithelial-mesenchymal transition (EMT) is a reversible biological process and an initial step in metastasis in which epithelial cells drop their epithelial characteristics (such as cell–cell adhesion, polarity, and the expression of the epithelial marker E-cadherin) and acquire mesenchymal properties (including encouraged overexpression of N-cadherin, vimentin, and fibronectin) along with altered expression of cell adhesion molecules and changes in the cytoskeleton [295]. Notably, EMT facilitates tumor metastasis by allowing cancer cells to separate from the primary site, invade adjacent tissues, enter the bloodstream, circulate throughout the body, and ultimately extravasate into distant organs [296, 297]. The activation of EMT is controlled by the EMT-inducing transcription factors (EMT-TFs), such as snail family Transcriptional repressor 1 (SNAI1), zinc finger e-box-binding homeobox (ZEB), and twist-related protein 1 (TWIST1) expression, along with microRNAs (miRNAs), as well as epigenetic and posttranslational regulators. These factors collectively mediate the protein expression implicated in cell–cell adhesion, cell polarity, cytoskeletal dynamics, and extracellular matrix (ECM) remodeling [298, 299]. From another investigation, TWIST1 is overexpressed in approximately 40% of NSCLC cases and connected with a more aggressive tumor phenotype, an increased risk of metastasis, and poorer patient prognosis [300]. Also, the molecular mechanisms of EMT activation involve multiple signaling pathways, such as receptor tyrosine kinases (RTKs), Wnt/β-catenin, TGF-β, Notch, and hedgehog, as well as adverse cellular conditions, such as inflammation and hypoxia, which play a crucial role in tumor progression and metastasis [301, 302], depicted in Fig. 4.

In NSCLC-related studies, researchers have found that the EMT process is linked to enhanced cancer cells’ plasticity, invasiveness, resistance to therapy, and poor prognosis [303–306]. Particularly, it has been shown that the downregulation of epithelial markers (such as E-cadherin and ZO-1) and the upregulation of mesenchymal markers (such as N-cadherin and vimentin) via the EMT process are critical for the invasiveness and metastatic potential of NSCLC cell lines [307]. For example, Ceteci et al. [308] demonstrated that, in a transgenic mouse model of ADC subtype carrying oncogenic C-Raf, either conditional deletion or dominant-negative expression of E-cadherin led to an increased vascular invasion, enhanced tumor growth, and progression to micrometastasis, which is mediated by nuclear translocation of β-catenin. In another view, Richardson et al. [309] demonstrated that vimentin-deficient transgenic mice (*LSL-Kras*^G12D^ and *LKB1*^fl/fl^) developed primary lung ADC with a tumor burden and growth pattern similar to wild-type mice. However, these vimentin-deficient mice exhibited a significantly reduced frequency of metastasis to the mediastinal lymph nodes and less focal invasion surrounding primary tumors compared to those with normal vimentin expression.

Several EMT-inducing TFs, including zinc-finger E-box-binding homeobox 1 (ZEB1) and TWIST, have also been clinically implicated in NSCLC metastasis [310–313]. For example, Chen et al. [314] demonstrated that the ZEB1 transcription factor alleviates miR-200-mediated repression of PD-L1 expression on NSCLC cells, which leads to intratumoral CD8^+^ T-cell immunosuppression and promotes mNSCLC. While not a classical EMT-TF, glioma-associated oncogene homolog 1 (GLI1) has been described to positively mediate SNAI1 expression by binding to its promoter and enhancing protein stability. This mechanism facilitates the activation of EMT, migration, invasion, and collectively promotes the metastasis of NSCLC [315].

Moreover, the direct role of EMT in regulating PD-L1 expression through epigenetic reprogramming has been correlated with immune evasion in NSCLC [316]. This mechanism involves demethylation of the PD-L1 promoter induced by TGF-β signaling and nuclear factor kappa B (NF-κB) activation, induced by tumor necrosis factor-alpha (TNF-α) expression [317]. Additionally, TGF-β is predicted to alter intracellular amino acid metabolism by inducing the prolyl 4-hydroxylase subunit alpha 3 (P4HA3) expression, supporting the characteristics of mNSCLC both in vitro and in vivo [318]. M2-type tumor-associated macrophages (TAMs) also secrete TGF-β, which further promotes Sox9 expression in NSCLC cells via the c-Jun/Smad3 pathway—thereby inducing EMT and increasing the migration of NSCLC cells [319]. Similarly, interleukin 6 (IL-6) released by M2-type TAMs enhances the cyclooxygenase-2 (COX-2) and prostaglandin E2 (PGE2) expression in cancer cells, which has been demonstrated in promoting the nuclear translocation of β-catenin and stimulating EMT in NSCLC cell lines [320].

Furthermore, an elevated presence of tumor-infiltrating CD4^+^ FOXP3^+^ Tregs has been described in patients with the 'mesenchymal' lung adenocarcinoma phenotype in comparison to those with an 'epithelial' lung adenocarcinoma phenotype [321]. This mesenchymal tumor phenotype is also associated with an increase in expression of immune checkpoint molecules, comprising B7 Homolog 3 (B7-H3), B and T lymphocyte attenuator (BTLA), CTLA-4, PD-1, PD-L1/2, and T-cell immunoglobulin and mucin-domain containing-3 (TIM-3), within the inflammatory TME [321]. Additionally, EMT status in circulating tumor cells (CTCs) has been correlated with upregulated PD-L1 expression [322] and development of therapeutic resistance by phenotypic switching in NSCLC [323].

Collectively, the EMT-driven axis of metastasis, immune modulation, and therapeutic resistance underscores its potential as a critical therapeutic target in the management of aggressive NSCLC phenotypes. Further exploration of EMT regulators and their signaling pathways promoting NSCLC metastasis, as summarized in Table 8, may provide novel strategies for improving NSCLC prognosis and treatment outcomes.

**Table 8:** Summary of key regulators reported for EMT-induced metastatic NSCLC

Mechanism	Factors	Remarks	Ref
**Cells**	CAFs	Enhance vimentin and reduce E-cadherin	[324]
		Release IL-6 and initiate JAK2/STAT3 signals	[324, 325]
		Secrete IL-22 and trigger PI3K/PKB/mTOR signals	[326–328]
		Secrete VCAM-1 and stimulate PKB and MAPK pathways	[329]
	TAMs	Upregulate α-B-Crystallin and regulate the ERK1/2/Fra-1/Slug pathway	[330]
	Th9 and Th17 lymphocytes	Released IL-9 and IL-17 induced gene expression for EMT transformation	[331]
	Schwann cells	CXCL5 interacts with CXCR2 to activate PI3K/PKB/GSK-3β/SNAI1-TWIST pathway	[332, 333]
**Signaling network**	FHL2/EMT	FHL2 induces EMT to enhance tumorigenesis	[334]
	LIX1L/EMT	LIX1L induces EMT to support cancer progression	[335]
	TRIM66/MMP-9	TRIM66 promotes MMP-9 levels to induce EMT and metastasis	[336]
	PHF5A/HDAC8/EMT	PHF5A increases HDAC8 levels to induce EMT	[337]
**Non-coding RNA**	CircCCT3/miR-107/Wnt/FGF7	Reduces miR-107 expression to induce Wnt/FGF7 axis, resulting in EMT to support invasion of tumor cells	[338]
	LINC00461/miR-4478/E2F1	Promotes E2F1 expression via miR-4478 sponging to induce EMT to cancer growth	[339]
	miR-224/HIF-1α/EMT	Reduces expression of SIRT3 to induce AMPK signaling which further suppresses mTOR to enhance HIF-1α expression in EMT induction	[340]
	LINC00673/miR-150–5p/EMT	Stimulates EMT via miR-150–5p sponging in improving cancer progression	[341]
	miR-21–5p	Exosomal miR-21–5p decreases apoptosis, improves M2 polarization of macrophages and induces EMT	[342]
	miR-155 miR-196a-5p	TAMs secrete exosomal miR-155 and miR-196a-5p for EMT development via negative regulating of RASSF4 expression	[343]
	LncRNA BCAR4/EMT	Increases cancer invasion properties via EMT induction	[344]
	FBXL19-AS1/EMT	Stimulates EMT to support advancing cancer invasion	[345]

### Cancer stem cells

**Fig. 5:**
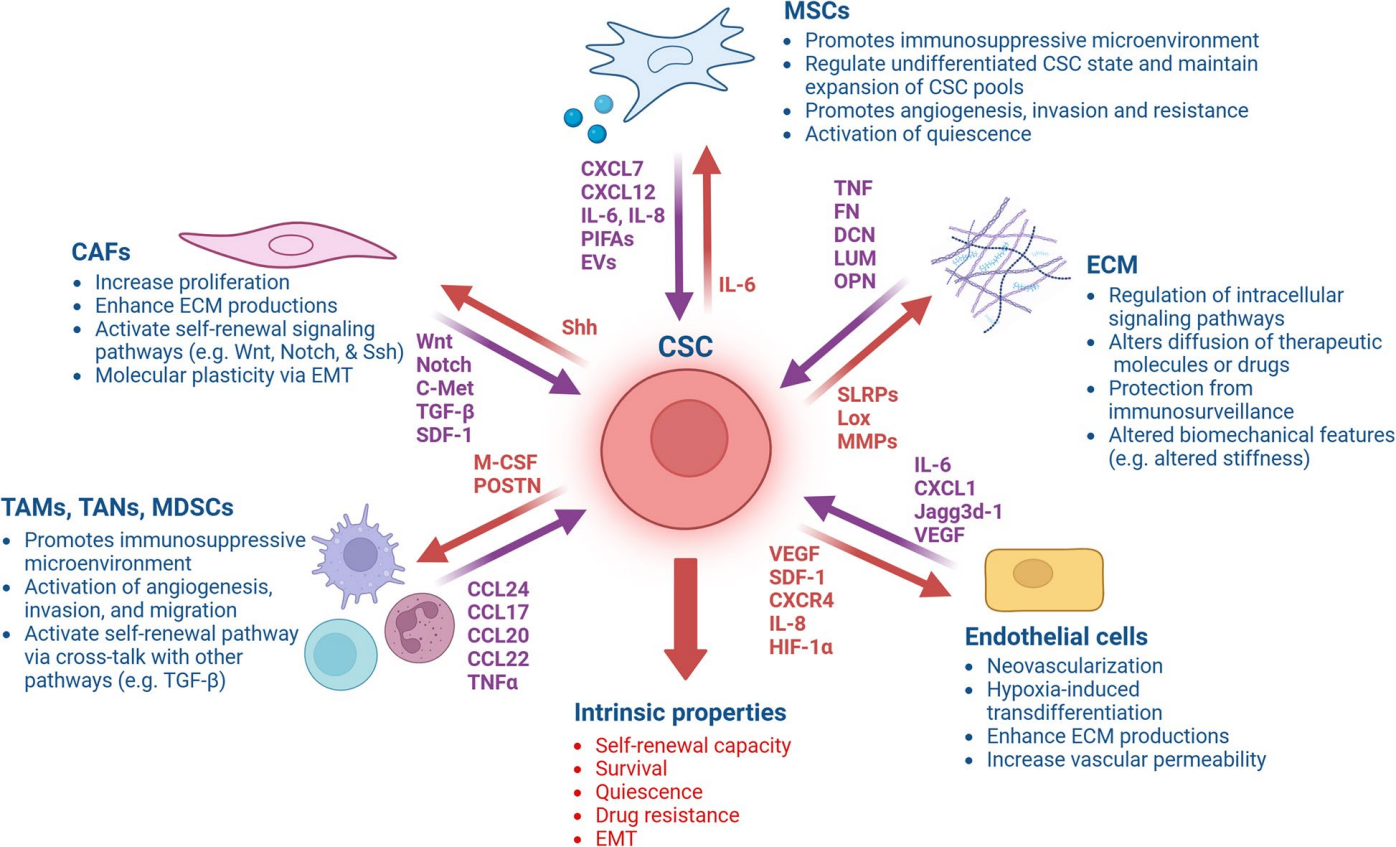
Cancer Stem Cells (CSCs) shows a complex interaction profile with the various components of the tumor microenvironment (TME), including cancer-associated fibroblasts (CAFs), tumor-associated macrophages (TAMs), tumor-associated neutrophils (TANs), myeloid-derived suppressor cells (MDSCs), mesenchymal stem cells (MSCs), endothelial cells, and the extracellular matrix (ECM). Various signaling molecules, including cytokines, chemokines, growth factors, and extracellular vehicles (EVs), mediate these interactions. CSCs receive regulatory signals from different TME components, including Wnt, Notch, c-Met, TGF-β, VEGF, SDF-1, IL-6, and IL-8, which facilitate tumor progression, immune evasion, and therapeutic resistance. The ECM plays a crucial role in regulating intracellular signaling pathways, altering drug diffusion, providing protection from immunosurveillance, and modifying the tumor's mechanical properties. The arrows represent the bidirectional communication between CSCs and the TME, contributing to tumor heterogeneity and disease progression. These dynamic interactions promote CSC intrinsic properties, including stemness, self-renewal, survival, angiogenesis, EMT, drug resistance, and quiescence, as illustrated based on insights from published literature [353]. Created in BioRender. Bharadwaj, S. (2025) https://BioRender.com/pe728gg

Cancer stem cells (CSCs) are depicted as heterogeneous populations, each with an exclusive functional role in the tumor [346, 347]. For instance, CSCs are accountable for raising growth, recurrence, and remote metastasis [348, 349]. Typically, CSCs depict specific immunological properties to produce gain resistance against chemotherapeutic drugs, as well as towards apoptosis-inducing immune effectors such as T cells or NK cells [350]. Accordingly, immune escape mechanisms are employed by CSCs, including impaired antigen presentation, negative regulation of MHC class I and II molecules, and secretion of immunosuppressive factors, to evade immune surveillance, sustain cancer progression, and facilitate cancer metastasis [351, 352]. A complex crosstalk of the CSCs with the microenvironment to support tumor growth and proliferation is illustrated in Fig. 5 [353].

In NSCLC cases, up to 63% of patients have been reported to exhibit histologic heterogeneity (more than one histologic subtype). This observation supports the hypothesis that lung cancer originates from a multipotent stem-like cell capable of differentiating into various cell types, leading to various observed histological patterns [354]. ​The first report of CSCs in NSCLC came from a mouse model with an inducible *KRAS*^G12D^ mutation, where a subpopulation of cancer cells was witnessed to demonstrate self-renewal, differentiation, and tumor regeneration capabilities [355]. Moreover, CSCs co-express C-X-C Chemokine receptor Type 4 (CXCR4) and ecto-5'-nucleotidase (CD73, also known as NT5E–5'-Nucleotidase, Ecto) were found augmented in LNM compared to primary NSCLC, suggesting that the cells with the potential to originate metastasis may carry a potential immunosuppressive activity [356]. Notably, the CXCR4 expression has been labeled as a major factor in preserving stemness and conferring metastatic potential of CSCs for NSCLC development [357]. Another study characterized CD133^+^ CXCR4^+^ metastasis-initiating cells (MICs) in NSCLC for potentially evading the immune surveillance through overexpression of PD-L1 and ectoenzymes CD73/CD38, which further facilitate the extracellular adenosine production, contributing to an immunosuppressive microenvironment [358].

To identify CSCs in lung cancer, specific biomarkers have been characterized, including ATP binding cassette subfamily G member 2 (ABCG2), aldehyde dehydrogenase (ALDH), CD166/Nanog homeobox (NANOG), CD133/prominin 1 (PROM1), CD44, CD117/KIT, and POU class 5 homeobox 1 (POU5F1, formerly known as OCT3/4) [359, 360]. For instance, analysis of patient-derived lung cancer cells revealed a subpopulation of ALDH^high^CD44^high^ cells characterized by enhanced self-renewal, clonogenicity, tumorigenicity, invasiveness, and resistance to chemotherapy [361]. Also, the majority of the human lung ADC samples were noted with an overexpression of Clara cell secretory protein (CCSP) and OCT4, typical features of bronchioalveolar stem cells (BASCs) [355, 362]. However, it is important to mention that, to date, no single biomarker is sufficiently robust and reproducible for detecting specific subsets of CSCs in NSCLC tumors. The key markers and cellular or molecular functions of CSCs identified in NSCLC are summarized in Table 9.

**Table 9:** Putative cancer stem cells markers reported in NSCLC

Class of biomarker	Name of biomarker	Description and cellular/molecular function of putative or identified CSC biomarkers in NSCLC	Ref
Cluster of differentiation (CD)	CD44	• Type I transmembrane glycoprotein (85–250 kDa) • Binds to variety of ligands on the cell surface, including hyaluronic acid • Trigger multiple cellular processes such as cell–cell signaling, cell growth and differentiation, cell adhesion and migration, angiogenesis, or cell survival • Associated with a more aggressive tumor phenotype, with the ability to metastasize and resist chemotherapy	[381, 382]
	CD117	• Type I transmembrane glycoprotein (approximately 145 kDa) • Activate tyrosine kinase growth factor receptor • Self-renewal and chemoresistance	[383, 384]
	CD133, also named Prominin 1 (PROM1)	• Cholesterol-binding five-fold transmembrane glycoprotein (97–120 kDa) • Essential to maintain CSCs characteristics such as tumor cell proliferation, migration, and invasion, and the ability to resist chemotherapy	[385–387]
	CD166	• Type I transmembrane glycoprotein (100–105 kDa) member of the immunoglobulin superfamily • Activate leukocyte cell adhesion molecule (ALCAM)	[388, 389]
Surface markers, not CD	CXCR4	• G protein-coupled seven-transmembrane protein (40–70 kDa) • Support chemokine receptor expression followed by enhanced invasiveness and metastasis • Support CSC self-renewal activity and tumorigenicity in vivo	[357, 390]
	ALDH	• Functioned through ALDH1A1 and ALDH3A1 isotypes • ALDH1 activity promotes CSCs features including proliferation, self-renewal, and differentiation, resistance to chemotherapy • ALDH3A1 overexpression drives cancer stem cell expansion, Impairing immune surveillance via enhanced PD-L1 expression	[391, 392]
	BMI-1	• A protooncogene of polycomb group family member • Represses the tumor suppressor phosphatase and tensin homolog (PTEN), and promotes cell proliferation	[393]
	EpCAM	• Single transmembrane protein (30–40 kDa) • Participates in cell–cell contact adhesion strength and tissue plasticity • Increased the metastatic capacity of tumor cells	[394]
	ABCG2	• Half transporter (approximately 72 kDa) composed of six transmembrane domains and only one ATP-binding domain • Transportation of xenobiotic compounds into the extracellular space and associated with cancer relapses and poor prognosis	[395]
Transcription factors	Oct-4, also known as POU5F1	• Member of the POU transcription factor family (16.4 kb) • Target *Fgf4*, *Utf1*, *Opn*, *Rex1/Zfp42*, and *Fbx15* genes, associated with chemotherapy resistance, cancer relapse, and worse outcomes	[396]
	Sox2	• Member of the high mobility group (HMG) box gene family (~ 2.4 kb) encoded by the sex-determining region Y-box gene • Regulate the transcription of the c-MYC, Wnt and Notch oncogenes and increase metastatic capacity through the FGFR-ERK1/2-SOX2 signaling pathway • Sox2 and Oct-4 enhance CD133 expression under hypoxia, promoting self-renewal and maintenance of lung CSCs	[397–399]
	Nanog	• DNA binding homeobox transcription factor (~ 150 kb) • DNA-binding homeobox transcription factor • Upregulated by Nr5a2 promoting CSCs properties and tumorigenesis in NSCLC	[400]

Where, *CD44* Cluster of Differentiation 44, *CD117* Stem Cell Factor Receptor (c-Kit), *CD133* Prominin-1 (PROM1), *CD166* Activated Leukocyte Cell Adhesion Molecule (ALCAM), *CXCR4* C-X-C Chemokine Receptor Type 4, *ALDH* Aldehyde Dehydrogenase, *BMI-1* B Lymphoma Mo-MLV Insertion Region 1 Homolog, *EpCAM* Epithelial Cell Adhesion Molecule, *ABCG2* ATP-Binding Cassette Sub-Family G Member 2, *Oct-4* Octamer-Binding Transcription Factor 4 (POU5F1); Sox2: SRY-Box Transcription Factor 2; Nanog: Homeobox Protein NANOG

In lung CSCs, developmental singaling pathways, such as Hedgehog, Wnt, and Notch pathways, play crucial roles in the regulation of key mechanisms, including self-renewal and differentiation [363–366]. Experimental studies on mouse and human airway basal stem cells (ABSCs) demonstrated that Notch signaling activation is essential for ROS to trigger ABSCs self-renewal [367]. In an in vivo mouse tumorigenesis model, IL-10 produced by TAMs was shown to promote stemness in lung cancer via the JAK1/STAT1/NF-κB/NOTCH1 pathway [368]. A large study by Westhoff et al. [369], involving tumor tissues from more than 400 NSCLC patients, reported an activation of the Notch pathway in approximately one-third of the cases. Likewise, Levina et al. [370] explained that NSCLC cells that survive cytostatic treatment typically showed stem cell-like characteristics and activation of the Wnt pathway. In another study, Chang and colleagues [371] established a direct relationship between mesenchymal reprogramming and stemness induction via the Wnt pathway in NSCLC. Similarly, Zhang et al. [372] reported that Gata6-regulated Wnt signaling is crucial for maintaining the balance between progenitor cell expansion and epithelial differentiation, a process essential for lung development and regeneration in a murine model. Regarding Hedgehog signaling, Shi et al. [373] and Tian et al. [374] demonstrated that Hedgehog signaling inhibition using Cyclopamine or Vismodegib (GDC-0449) significantly blocked the proliferation of the side population (SP) in a pulmonary NSCLC cell line. Furthermore, the discussed embryonic signaling pathways have also been demonstrated to maintain CSCs, likely reflecting their origin from tissue-specific progenitor cells [365]. In addition, the tumor suppressor gene *PTEN* has been consistently demonstrated to mediate the regulation of CSCs in NSCLC. For instance, in a murine model with constitutive PTEN loss, epithelial cells at the bronchioalveolar duct junction proliferated through the PI3K/AKT/hTERT pathway, resulting in the formation of uniform masses [375]. Moreover, extracellular signal-regulated kinase (ERK) inhibition also sponsors cancer cell dedifferentiation and expands the CSC population in NSCLC [376]. Interestingly, CSCs isolated from NSCLC cell lines have been shown to differentiate into CD2^+^ Th17-like immune cells, suggesting that a subset of immune cells within the TME may derive from CSCs. These CD2^+^ Th17-like immune cells secrete cytokines IL-17A and IL-22, which are known to promote proliferation and metastasis in NSCLC [326, 377].

Moreover, metastatic cancer stem cells (mCSCs), a subset of CSCs, can respond to signals from the ECM of distant organs, enabling them to escape the primary tumor and contribute to metastasis [378]. Activation of EMT promotes the invasive phenotype of MCSCs and facilitates their participation in the metastatic cascade. Several EMT-TFs are key regulators of stemness in CSCs, which acts as signaling molecules to influence the cell behavior and promote metastasis [379]; thus, EMT is closely linked to the acquisition of tumor cell stemness [301]. Furthermore, constitutively activated *EGFR*-mutant cancer cells have been found to overexpress spalt-like Transcription factor 4 (SALL4)—a member of the zinc finger transcription factor which fuses with POU5F1, Sox2, and KLF4—resulting in the maintenance of CSC properties, including pluripotency gene expression and spheroid formation in NSCLC [380].

Conclusively, CSCs play a pivotal function in NSCLC metastasis and therapy resistance due to their immunosuppressive properties and reliance on key developmental signaling such as Hedgehog, Notch, and Wnt pathways. Despite advances in identifying CSC biomarkers, challenges persist in establishing robust and reproducible biomarkers for effectively targeting CSCs. Besides, addressing CSC heterogeneity and their interactions with the immune cells in the TME is also important for developing innovative therapeutic approaches to transform NSCLC treatment, reduce recurrence, and improve overall survival in cancer patients.

### Non-coding RNAs

Non-coding RNAs (ncRNAs) do not encode proteins but perform essential jobs in controlling protein expression and function [401–403]. Several types of ncRNAs—comprising microRNAs, long non-coding RNAs, and circular RNAs—have been reputed to influence cell growth, proliferation, and metabolism via diverse mechanisms [403, 404]. A list of key ncRNAs implicated in NSCLC metastasis is summarized in Table 10.

**Table 10:** Functional roles of ncRNAs involved various stages of NSCLC metastasis

ncRNAs	Molecules	Functions	Ref
miRNAs	miR-21	Promoting macrophage polarization, EMT, and accelerated ERK/STAT3 signaling	[431]
	miR-19b-3p	Promote M2 macrophage-derived exosomal LINC00273 mediated Hippo/YAP pathway by LATS2 ubiquitination	[432]
	miR-210	Help CAFs release FGF2, VEGF and activate JAK2/STAT3 pathway	[433]
	miR-411-5p/3p	Target *SPRY4*, *TXNIP* and activate EGFR, PKB	[434]
	miR-421	Target *HOPX* and activate Wnt/β-catenin	[435]
	miR-183-5p	Decrease the expression of PTEN and p53	[436]
	miR-629-5p	Reduce the expression of PPWD1 and CELSR1	[437]
	miR-196b-5p	Decrease the expression of GATA6 and TSPAN12	[438]
circRNAs	circSATB2	Interact with miR-326 and promote the level of *FSCN1*	[422]
	circFARSA	increase ubiquitination and degradation of PTEN and activate the PI3K/AKT signaling pathway	[439]
	circRNA-001010	Modulating the inhibitory effect of miR-5112 on oncogene CDK4	[440]
lncRNAs	PCAT6	Combine with miR-330-5p	[441]
	JPX	Combine with miR-33a-5p and increase the level of *Twist1*	[442]
	HOXA-AS2	Enhance the expression of IGF2	[443]
	MetaLnc9	Interact with PGK1 to activate PKB/mTOR and NONO to activate CRTC	[444]
	JHDM1D-AS1	Combined with DHX15	[445]

MicroRNAs (miRNAs or miR) are short, single-stranded, and endogenous ncRNAs that control gene expression post-transcriptionally as well as participate in cellular and physiological functions, including cell proliferation and renewal, repair, and neoplastic transformation, reviewed elsewhere [405]. In NSCLC, exosomes secreted by M2-type TAMs were found to contain miR-155 and miR-196a-5p, which target *RASSF4* to modulate RASSF4 expression, promoting EMT and migration, thereby supporting NSCLC metastasis [343]. Similarly, miR-942, secreted via exosomes from M2-TAMs, was shown to regulate FOXO1 expression and trigger the Wnt/β-catenin pathway in ADC-specific NSCLC cells [406]. In another study, macrophages were found to consume exosomal miR-181 secreted by NSCLC cells, which leads to their transformation into M2-type TAMs via activation of the JAK2/STAT3 signaling pathway [407]. Moreover, miRNAs also affect CSCs by post-transcriptionally regulating genes associated with stemness in NSCLC [360, 408, 409]. For instance, miR-150 was demonstrated to encourage cell invasion and metastasis in NSCLC by downregulation of forkhead box O4 (FOXO4), a negative regulator of cell proliferation [410]. This downregulation leads to the enhanced activation of the anchorage-independent growth, migration, and invasion of NSCLC cells, partially through activation of EMT via the NFκB/SNAI1/YY1/Raf kinase inhibitor protein (RKIP) signaling pathway [410]. In another study, using in vitro and in vivo models, miR-522-3p was also identified as a key modulator of NSCLC-specific brain metastasis, while miR-522-3p/TNS1 was demonstrated to alter blood–brain barrier (BBB) permeability [411].

Long non-coding RNAs (lncRNAs), a subgroup of ncRNA molecules longer than 200 nucleotides, participate in the modulation of several biological processes, such as cell division, cell differentiation, and epigenetic regulation [412, 413]. Interestingly, recent studies have demonstrated an essential role of lncRNAs in the invasion and metastasis of NSCLC cells via the regulation of the EMT process. In this context, lncRNA FBXL19-AS1 was demonstrated to accelerate the NSCLC proliferation and metastasis by modulation of the EMT process [345]. Other reports have also documented the impact of lncRNA on TAMs to support mNSCLC. In this context, Han et al. [414] discovered that lncRNA in or outside TAMs could also substantially manipulate the polarization of TAMs while indirectly encourages tumor progression [414]. Similarly, Lin et al. [415] showed that lncRNA HOXC-AS2 could bind to STAT1 in TAMs, actively control the STAT1/CIITA and STAT1/SOCS1 pathways along with polarization of TAMs to promote NSCLC progression. Also, lncRNA PKMYT1AR was demonstrated to inhibit β-catenin protein ubiquitination, enabled via the PKMYT1AR/miR-485-5p/PKMYT1 pathway, thereby sustaining CSC properties and promoting advanced NSCLC [416].

Circular RNAs (circRNAs), a type of non-coding RNA (ncRNA), are generated through and possess a stable, ring-like structure that resists degradation by RNA enzymes [417] and serve as an miRNA sponge to mediate downstream gene regulation [418]. In the context of NSCLC, upregulation of circRNAs has been demonstrated in lung cancer and is associated with NSCLC metastasis. Mechanistically, circRNAs are demonstrated to encourage mNSCLC by adsorbing miRNAs to control the expression of downstream pathways or modifying the transcription of target genes [419, 420]. For instance, circ_0008003 and exosomal circSATB2 were demonstrated to regulate zinc finger protein 281 (ZNF281) and fascin actin-bundling protein 1 (FSCN1), respectively, by sequestering miR-488 and miR-326, thereby promoting proliferation and metastasis of NSCLC cells [421, 422]. Likewise, circSATB2 (circRNA special AT-rich sequence‐binding protein 2) was predicted to bind with miR-326 to modulate the FSCN1 expression, which further supports the cell proliferation, migration, and invasion in NSCLC [422]. In another study, exosomal circ_0001715 was demonstrated to promote cell growth and metastasis in NSCLC via the miR-1249-3p/FGF5 axis [423]. In contrast, ADC cell-derived exosomal circ_0001715 were reported to promote tumor cell proliferation and metastasis by increasing M2-type TAMs polarization via the miR-205-5p/TREM2 axis [424]. In another study, circZNF451 was found to induce TAM polarization in ADC-specific NSCLC to reshape the TME [425] while high circ_0005909 expression was substantially associated with TNM stage and distant metastasis in NSCLC patients [426]. Similarly, circ_0006692, which is overexpressed in lung cancer and promotes cancer progression via the miR-205-5p/CDK19 axis, was positively associated with TNM stage and invasion of the lung basal layer [427].

Recently, a novel class of ncRNAs called P-element induced wimpy testis (Piwi)-interacting RNAs (piRNAs) has gained attention for its function in tumorigenesis. In this context, Li et al. and co-workers [428] established that an overexpression of piR-651 in A549 NSCLC cells led to an increased cell viability and metastasis. Also, they identified a substantial association of cyclin D1 (CCND1) and cyclin-dependent kinase 4 (CDK4) expression with an upregulation of piR-651, suggesting piR-651 can either trigger a highly proliferative or self-renewal potential in NSCLC cells [428]. Another important class of newly discovered ncRNA includes small nucleolar RNAs (snoRNAs; divided into two categories according to their structural basis: H/ACA snoRNAs (SNORAs) and Box C/D snoRNAs (SNORDs), which guide rRNA modification and have recently been demonstrated for regulatory functions in tumorigenesis [429]. For instance, SNORD88C promotes 2′-O-methylation of 28S rRNA at the C3680 site, resulting in enhanced translation of SCD1 of stearoyl-CoA desaturase 1 (SCD1), a key lipogenic enzyme involved in monounsaturated fatty acid (MUFA) synthesis. An overexpression of SCD1 inhibits autophagy by modulating lipid peroxidation and mTOR signaling, thereby facilitating the migration and invasion of NSCLC [430].

In conclusion, ncRNAs play pivotal functions in influencing the tumor microenvironment by modulating gene expression, EMT, CSCs, and immune interactions, thereby contributing to tumor progression and therapeutic resistance. An innate understanding of their production and molecular mechanisms offers promising avenues for the development of novel diagnostic biomarkers and targeted therapies designed to improve overall survival outcomes in NSCLC patients.

### Immunosuppressive tumor microenvironment

The tumor immune microenvironment (TIME) is a vastly ambiguous environment, mainly comprising inflammatory cells, cancer-associated fibroblasts (CAFs), and ECM, and is directly associated with multiplex biological events in tumors [446]. Importantly, the cellular interactions between tumor cells and immune cells, including myeloid-derived suppressor cells (MDSCs), tumor-infiltrating lymphocytes (TILs), and tumor-associated macrophages (TAMs), essentially subsidize the generation of immunosuppressive TME, resulting in deficient immunosurveillance and reduced clinical outcomes [447–449]. Recent studies have also deciphered stage-dependent immune cell infiltration [450, 451], suggesting that the TME is an essential factor in promoting lung carcinogenesis. Supporting this, a recent study established a higher proportion of immunosuppressive CD36^+^CD8^+^ T cells in NSCLC tissues compared to non-tumor tissues, which was substantially linked to the TNM stage [452]. Additionally, immunosuppressive cells, including MDSCs, TAMs, and CAFs, can smoothly acclimate to a nutrition-deprived TME due to their metabolic flexibility, and after adaptation, they habitually contribute to tumor immune evasion [453]. Thus, TIME significantly influences tumor growth, immune escape, metastasis, therapeutic response, and patient survival [454, 455].

Like other tumor types, NSCLC can initiate and sustain an immunosuppressive TME favorable to tumor growth [456–458]. For example, chronic inflammatory environments are predicted to alter or deviate the differentiation of immune cells in lung cancer, which further reduces the antitumor response and promotes resistance development to ICIs [459]. In addition, lung tumor cells may release immune suppressive cytokines, including interleukin (IL)−10 and transforming growth factor-β (TGF-β), to evade immune surveillance [460]. Moreover, the high ability of MSCs or metastasis-initiating cells (MICs) to evade immune surveillance and support the association between C-X-C Chemokine receptor type 4 (CXCR-4) pathway and immunosuppressive phenotype induction has been demonstrated in CSCs [356]. In another study, depletion of C-X-C motif Chemokine 5 (CXCL5 or ENA78) resulted in tumor angiogenesis inhibition, which further decreased the tumor progression and metastasis in NSCLC models [461]. Likewise, CD147-K148me2-mediated enhanced CCL5 expression in NSCLC cells was detected to promote intercellular crosstalk between tumor cells and M2-TAMs via the CCL5/CCR5 axis, supporting M2-TAMs infiltration in NSCLC and tumor progression [462]. Furthermore, functional inhibition of CXCR4 in tumor cells was discovered to effectively inhibit the immunosuppressive role of MICs by restoring T cell proliferation and IFNγ expression, and partly by blocking TAM polarization [356]. Therefore, the inhibition of abnormal intercellular crosstalk in the TME has been projected as a potential strategy to reverse the immunosuppressive TME and enhance the anti-tumor immune response in cancer patients [462]. Hence, this section provides a summary on the functional contribution of various immune resident cells within the TME that indirectly or directly promote its immunosuppressive characteristics.

### Tumor-associated immunosuppressive cells

#### Cancer-associated fibroblasts

**Fig. 6:**
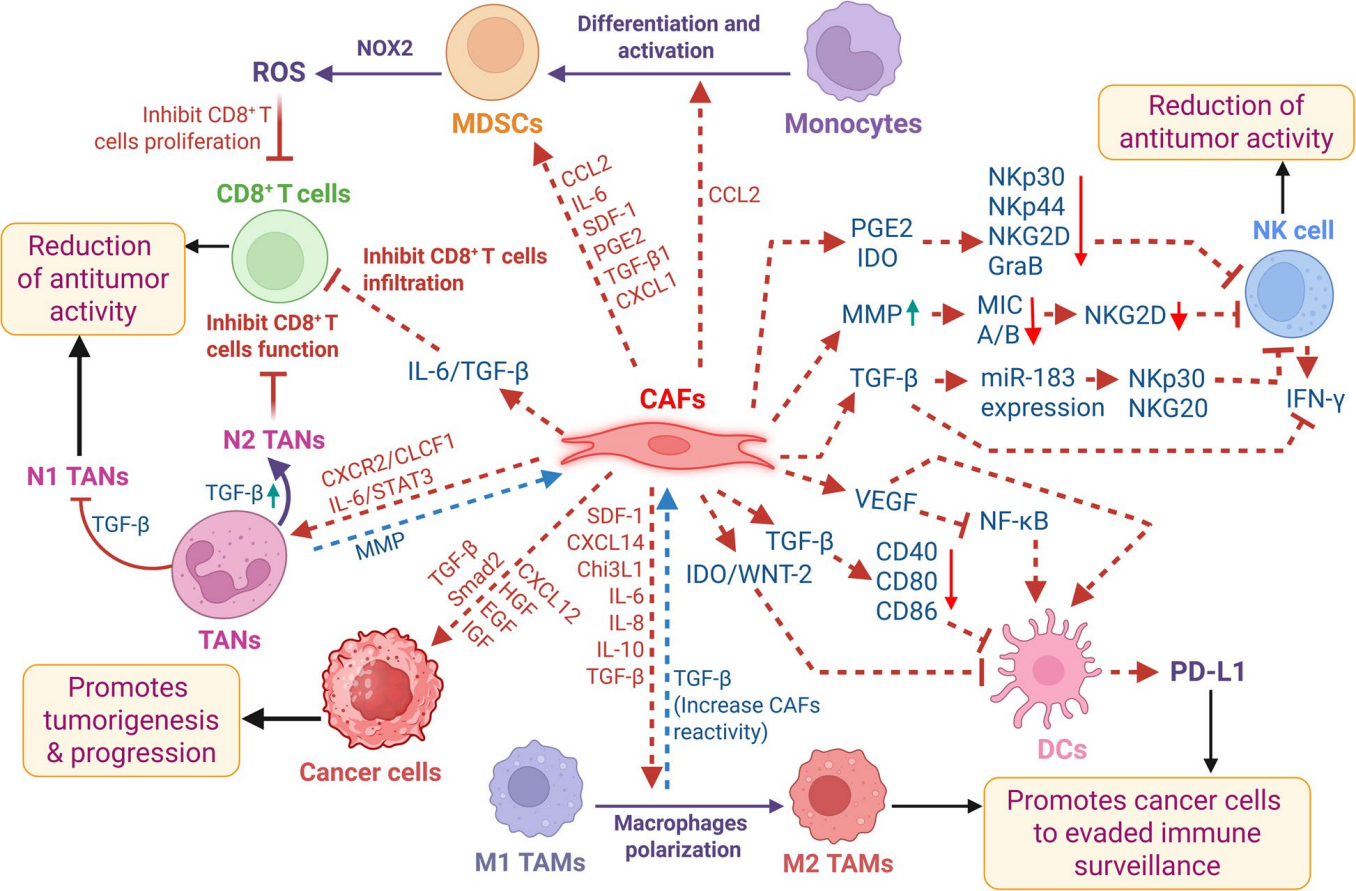
Schematic illustrating the crosstalk between cancer-associated fibroblasts (CAFs), cancer cells, and major immune cell groups within the tumor microenvironment (TME). CAFs interact with various immune cells—including tumor-associated macrophages (TAMs), tumor-associated neutrophils (TANs), dendritic cells (DCs), myeloid-derived suppressor cells (MDSCs), natural killer (NK) cells, and CD8⁺ T cells—to promote tumor development through the secretion of cytokines, chemokines, and other effector molecules such as TGF-β, IL-6, CCL2, and CXCL2. Notably, TAMs, TANs, and mast cells (MCs) reciprocally contribute to the formation of an immunosuppressive circuit by activating CAFs and enhancing their tumor-supportive functions [476, 477]. Created in BioRender. Bharadwaj, S. (2025) https://BioRender.com/3o71yeo

Fibroblasts are the most common and heterogeneous population of stromal cell types in solid tumors [463, 464], where they are designated as cancer-associated fibroblasts (CAFs). In tumors, CAFs are commonly identified based on α-smooth muscle actin (α-SMA) expression, “myofibroblastic” phenotype (myCAFs), analogous to that monitored in wound healing [465]. CAFs are crucial in cell proliferation, migration, and invasion [466, 467]; promoting angiogenesis [468]; depositing and remodeling the ECM [469]; and reshaping the immunosuppressive TME by dampening T cell reactions [470, 471]. A crosstalk between CAFs and other immune cells in TME resulting in mNSCLC is depicted in Fig. 6**.**

In NSCLC, discrete functional subsets of transcriptomically distinct CAF phenotypes, such as high desmoplastic CAFs (HD-CAFs) and low desmoplastic CAFs (LD-CAFs), have been identified [472–474]. For instance, HD-CAFs exhibit a higher rate of invasion and collagen matrix remodeling in comparison to LD-CAFs [474]. Furthermore, CAFs-secreted interleukin-22 (IL-22) has been shown to promote NSCLC proliferation and metastasis by mediating the PI3K/Akt/mTOR signaling pathway [326]. CAFs also prevent the death of NSCLC cells under serum-free conditions and support their tumorigenesis by releasing hepatocyte growth factor (HGF) in vivo [475]. In another study, stromal integrin alpha-11 beta-1 (ITGα11β1) expression was reported to mediate the transformation of normal fibroblasts (NFs) into CAFs [476], while overexpression of ITGα11β1 complex in CAFs was demonstrated to promote the metastasis of NSCLC cells by controlling collagen cross-linking and the regulation of fibrillar collagen matrices [476]. Additionally, high stromal integrin alpha-11 (ITGα11) expression in CAFs was found to promote their interaction with cancer cells, thereby increasing the metastatic potential of NSCLC cells [477, 478].

Furthermore, the role of glycosylation and epigenetics in CAFs has also been reported in the promotion of metastasis in NSCLC cells. For instance, Fengzhou Li et al. [479] showed that α1,6-Fucosyltransferase (FUT8), which catalyzes core fucosylation of N-glycans during glycoprotein biosynthesis, regulates proliferation and invasiveness of CAFs in NSCLC by amending EGFR core fucosylation in vitro and in vivo [479]. Similarly, CAFs were reported to enhance the metastatic potential of NSCLC cells through an N6-methyladenosine (m^6^A) modification-dependent regulatory mechanism, with methyltransferase-like 3 (METTL3) mediating m^6^A modification in NSCLC cells [480]. Besides, exosomal miR-210 secreted by CAFs was shown to target UPF1 to activate the PTEN/PI3K/AKT pathway, thereby promoting NSCLC metastasis via EMT induction [481]. Additionally, CAFs have been demonstrated to secrete high mobility group box 1 (HMGB1), which enhances EMT through NF-κB signaling in NSCLC [482].

In summary, the diverse functional subsets of CAFs in NSCLC, along with their ability to remodel the extracellular matrix, secrete key signaling molecules, and regulate glycosylation and epigenetic modifications, underscore their vital role in tumor progression and metastasis. A deeper understanding of CAFs’ phenotypes and their interactions with NSCLC cells could offer novel therapeutic strategies for targeting CAF-driven mechanisms in NSCLC treatment.

#### Dendritic cells

Dendritic cells (DCs) are a major class of antigen-presenting cells (APCs) and actively participate in T cell activation and differentiation, which is a crucial step in commencing anti-tumor immune responses [483]. Typically, DCs display immune responses based on their maturation state, where mature DCs orchestrate modulation of immune responses while immature DCs encourage immune tolerance [484]. Notably, the functional role of DCs can be corrupted by tumors.

In the context of NSCLC, tumor-derived DCs exhibit overexpression of the T cell co-inhibitory molecule B7-H3, which belongs to the programmed death ligand (PD-L) family, and show reduced stimulation of T cell proliferation compared to controls [485]. This event may render DCs immunosuppressive and impair their ability to stimulate antitumor T cells, thereby contributing to an immunosuppressive TME formation [485]. Furthermore, overexpression of B7-H3, detected in cell lines and tumor specimens, was significantly linked to LNM in NSCLC patients [486]. Additionally, high levels of circulating B7-H3 were correlated with tumor stage, nodal involvement, and distant metastasis in NSCLC [487]. In a recent study, the immunosuppressive phenotype of plasmacytoid DCs (pDCs), a subset of DCs, was identified based on the overexpression of IFN-α [488], and was discovered to be higher in the peripheral blood of patients with advanced stages of NSCLC [489]. Additionally, a high number of immunosuppressive pDCs with an overexpression of CD33 and PD-L1 were observed in NSCLC tissues compared to Healthy tissues, which produced substantial amounts of interleukin 1α (IL-1α) to promote cancer cell proliferation [490].

Conclusively, targeting the immunosuppressive mechanisms in DCs or reversing DC dysfunction could enhance anti-tumor immunity, offering a promising avenue for immunotherapeutic strategies in mNSCLC.

#### Myeloid-derived suppressor cells

Myeloid-derived suppressor cells (MDSCs) are a heterogeneous population of immature cells comprising myeloid progenitor cells, immature DCs, immature macrophages, and immature granulocytes, all of which exhibit potent immunosuppressive activity in the TME [491, 492]. Like other immune cell types, MDSCs can be further categorized into monocytic MDSCs (M-MDSCs; CD11b^+^Ly6G^−^Ly6C^hi^) and polymorphonuclear MDSCs (PMN-MDSCs; CD11b^+^Ly6G^+^Ly6C^low^) [493, 494]. Interestingly, each subtype of MDSCs adopts a distinct mode of action within tumor tissues to exert their immunosuppressive effects and facilitate tumor progression and metastasis [491, 494–496].

In NSCLC cases, a higher number of circulating M-MDSC expressing C–C Chemokine receptor type 5 (CCR5) have been identified in the peripheral blood of patients compared to healthy donors. Additionally, the incidence of both M-MDSCs and PMN-MDSCs was more prevalent in NSCLC tissues than in peripheral blood [497]. Importantly, MDSCs are characterized as a major group of immune precursor cells in NSCLC that directly or indirectly contribute to the formation and maintenance of the immunosuppressive TME [498, 499]. Mechanistically, MDSCs secrete various immunosuppressive molecules and induce an overexpression of inhibitory checkpoints in NSCLC patients. This results in the differentiation and development of Tregs, which further impedes the homing and trafficking of tumor-specific T cells into the TME [499]. For example, overexpression of Arg-1 and iNOS in MDSCs has been linked to the suppression of CD8^+^ T cell proliferation in NSCLC [499, 500]. Similarly, MDSCs with an overexpression of ectonucleotidases CD39 and CD73, which turn ATP into adenosine, were detected to promote an immunosuppressive TME in NSCLC patients [501, 502]. Moreover, MDSCs can also secrete exosomes that inhibit anti-tumor immune responses and support NSCLC metastasis [503–505]. High infiltration of MDSCs in tumors has also been associated with disease progression and the promotion of distant metastasis [506, 507]. In this context, MDSCs have been reported to activate the AKT and ERK pathways, facilitated by the overexpression of chemokines such as CCL11, which further promote invasion, EMT, and metastasis in NSCLC [508]. Notably, the EMT process mediated by MDSCs is associated with Treg recruitment, reduced infiltration of CD4^+^ and CD8^+^ T cells, and T cell exhaustion in the TME [509, 510].

Conclusively, different subtypes of MSDCs appear to adopt distinct mechanisms to promote NSCLC metastasis. As limited studies have explored the role of tumor-infiltrating MDSCs in NSCLC, the identification of regulatory factors in MDSCs could provide valuable insights into the immunosuppressive TME associated with mNSCLC.

#### Tumor-associated macrophages

Tumor-associated macrophages (TAMs) constitute the general population of innate immune cells in the TME that perform multiple tumor-supporting functions during all stages of carcinogenesis, from cancer onset to post-therapeutic metastatic relapse [511, 512]. In NSCLC, TAMs apply both pro- and antitumor effects through their two polarizing phenotypes, M1 (inflammatory) and M2 (anti-inflammatory), within the TIME. Interestingly, recruited macrophages in NSCLC are polarized into the M2 phenotype at the time of tumor initiation [513]. Accordingly, infiltrated M2-type TAMs are primarily predominant in the TIME and characterized by specific markers, such as CD163, CD204, and MARCO [514], as well as positively correlated with advanced stages of NSCLC [406, 515–517]. For example, a high density of M2-type TAMs has been demonstrated in mNSCLC tissues [343]. Another study predicted that M2-type TAMs enhance the CXCL1 and COL6A1 expression to advance the NSCLC metastasis via an NF-κB/PP2Ac positive feedback loop signaling pathway [518]. Similarly, TAMs have been reported to encourage cancer cell migration via exosomal signaling [519, 520]. In support, exosomal microRNA (miR)−19b-3p has been identified to accelerate the polarization of M2-type TAMs, thereby fostering NSCLC metastasis [432].

Moreover, TAMs constitute an important source of cytokines in the TIME [521]. Specifically, M2-type TAMs were characterized for the secretion of immune-modulating interleukins (IL-6, IL-8, and IL-10), which creates an immunosuppressive TME favorable for NSCLC growth and metastasis [522–524]. M2-type TAMs were also detected considerably in dysregulating the Tregs’ function via IL-10 signaling to promote immunosuppressive TME in NSCLC [525]. Furthermore, a recent study documented that NSCLC cells can educate TAMs to express MARCO and acquire an immunosuppressive phenotype by secreting IL-37, which diminishes the cytotoxic T cells and natural killer (NK) cells activation, as well as their tumor-killing ability [526]. Likewise, M2-type TAMs were found to upregulate CRYAB (alpha-crystallin B chain, a member of the small heat shock protein superfamily) expression on Lung cancer cells and activate the extracellular regulated kinase 1/2 (ERK1/2)/Fos-related antigen 1 (Fra-1)/Slug pathway linked to the EMT process, resulting in an enhanced NSCLC cell invasion and migration [330]. Guo et al. [330] similarly discovered that M2-type TAMs enhance the expression of α-B-Crystallin and activation of the ERK1/2/Fra-1/slug signaling, facilitating the EMT to advance the malignant mNSCLC. The same study also reported that high intertumoral CRYAB expression was associated with TNM stage and late-stage NSCLC patients [330].

Furthermore, TGR5 (also known as GPBAR1) was found on infiltrated TAMs, where it affects anti-tumor immunity by contributing to TAM-mediated blockage of CD8^+^ T cell functions and overall survival in NSCLC patients [527]. In another study, immunosuppressive triggering receptor expressed on myeloid cells 2 (TREM-2), a glycosylated receptor belonging to the immunoglobulin superfamily (IgSF) [528], expressed by TAMs phenotype was also detected in clinical specimens of NSCLC patients and linked to T cell function inhibition, including diminished anti-tumor activity of CD8^+^ T cells and enhanced differentiation toward FOXP3^+^ Tregs [529]. Importantly, high infiltration of immunosuppressive TREM-2^+^ TAMs has been substantially associated with advanced tumor progression and suboptimal ICB responsiveness in NSCLC patients [529].

In a recent comparative study between nivolumab-sensitive and nivolumab-resistant NSCLC patients, the latter exhibited a high infiltration of M2-type TAMs and a lower density of CD8^+^ T and NK cell infiltration in primary tumor tissue [530]. M2-type TAMs also showed upregulated METTL3 expression in NSCLC cells, which is suggested to produce an enhanced resistance against immune cells [530]. Additionally, M2-type TAM accumulation was significantly correlated with integrin alpha-V beta-3 (ITGαVβ3) expression on tumor cells, which directs tumor progression and therapeutic resistance in NSCLC [531, 532].

Overall, M2-type TAMs decisively contribute to NSCLC progression and metastasis by promoting immune evasion. Therefore, understanding the specific cellular signaling pathways that drive polarization from the immunostimulatory (M1) to the immunosuppressive (M2) TAM phenotype, and subsequent adopted pathways, could enhance our understanding of NSCLC metastasis.

#### Regulatory T Cells

Regulatory T Cells (Tregs), an immunosuppressive subgroup of CD4^+^ T lymphocytes, are characterized by an overexpression of the TF, forkhead box protein 3, and the interleukin 2 alpha (IL-2α) receptor [533, 534]. Typically, tumor-associated Tregs occupy approximately 10%–50% of CD4^+^ T cells in tumors, which is profoundly greater than that residing in the peripheral blood of healthy individuals (2%–5%) [535, 536]. Specifically, Tregs impose immunosuppressive function through contact-independent mechanisms (such as secretion of IL-1, IL-10, IL-35, galectin-1, prostaglandin E2, and TGF-β) and contact-dependent mechanisms (involving overexpression of CTLA-4, PD-1, PD-L1, lymphocyte-activation protein-3 (LAG-3), and neuropilin 1 (NRP1)) [537–540].

Notably, in murine models of NSCLC (lung ADC subtype), Tregs were identified to inhibit the CD8^+^ T cell-facilitated anti-tumor immunity [541]. However, depletion of Tregs at early stages of tumorigenesis was associated with increased tumor cell death and elevated levels of granzyme A, granzyme B, perforin, and IFN-γ in infiltrating CD8⁺ T cells [541]. In another in vivo model’s investigation, it was revealed that Tregs stimulates activation-induced cell death (AICD) of T lymphocytes, which further favors LNM via metabolic reprogramming of metastatic cells [542]. Tregs are also linked to an overexpression of angiogenic and metastatic potentiator cyclooxygenase-2 (COX2), where preeminent numbers of intratumoral FoxP3^+^ lymphocytes are connected with high intratumoral COX2 expression [543, 544]. Additionally, a study on mouse models of Lewis lung carcinoma demonstrated that Tregs can inhibit NK cell–mediated cytotoxicity in a TGF-β–dependent manner [545].

Similarly, an increase in number of Tregs and activity was detected in multiple tissues of NSCLC patients, such as LNM and the peripheral blood, and was profoundly associated with tumor staging and recurrence frequency of NSCLC metastasis [546–549]. For instance, a clinical study on 23 patients with NSCLC observed that the number of Tregs increases in the peripheral blood with advancement in the tumor stage and was mostly located in NSCLC metastatic regions [546]. Moreover, tumor cells from NSCLC patients with advanced disease stages and LNM exhibit higher secretion of TGF-β compared to those from early stages [550]. This results in an enhanced TGF-β production to promote tumor infiltration and the immunosuppression function of Tregs by upregulating the expression of inhibitory molecules, such as B7H1 and glucocorticoid-induced tumor necrosis factor receptor-related ligand (GITRL), on the surface of APCs [551]. Moreover, highly proliferative tumor necrosis factor receptor 2 positive (TNFR2^+^) Tregs with overexpression of CTLA-4 molecules were also detected in the peripheral blood and pleural effusions, and were assumed to mediate stronger immunosuppression than TNFR2^−^ Tregs in NSCLC patients [552, 553]. Mechanistically, the constitutive high expression of CTLA-4 on the surface of Tregs was directly correlated with the inhibition of T cell proliferation in NSCLC tumors [554, 555]. Similarly, Treg signaling was found to trigger the GATA binding protein 3/Nitric oxide synthase 2 (GATA3/NOS2)-associated immunosuppression by inhibition of the stimulator of interferon genes (STING) pathway—which blocks CD4^+^ T infiltration—in NSCLC tissues, thereby promoting pre-metastatic niche development and increasing lung metastatic burden [542].

Altogether, these discoveries underscore the significance of Treg infiltration into tumors in promoting immunosuppression, tumor progression, and metastasis in NSCLC. However, precise molecular mechanisms behind their recruitment and distinct functions in the immunosuppressive TME remain poorly defined in NSCLC. Addressing these gaps—particularly their heterogeneity and interactions within the TME—could advance immunotherapy for primary and advanced NSCLC cases.

#### T Helper 17 cells

T Helper 17 (Th17) cells, a subset of effector T helper cells (CD4^+^ T helper cells), exhibit a distinct phenotype compared to Th1 and Th2 cells and are identified based on the expression of TFs retinoic acid receptor-related orphan receptor γt (RORγt)/retinoic acid receptor-related orphan receptor C2 (RORC2), and retinoic acid receptor-related orphan receptor α (RORα) [556, 557]. These immune cells actively mediate the immune response by releasing the highly versatile proinflammatory cytokine interleukin-17 (IL-17), which further prompts the secretion of inflammatory mediators by epithelial cells, fibroblasts, or macrophages [558, 559]. Notably, IL-17 secretion enables cancer cells to evade immune surveillance within the TME [556, 557] and also promotes the development of EMT to advance the NSCLC migration and metastasis [331]. Also, IL-17 has been shown to enhance angiogenesis by encouraging cancer cells to release vascular endothelial growth factor (VEGF) via the signal Transducer and activator of Transcription 3 (STAT3)/girdin-like protein (GIV) signaling pathway in NSCLC tissue [560].

In summary, Th17 cells via IL-17 drive inflammation, immune evasion, EMT, metastasis, and angiogenesis in NSCLC, highlighting their crucial role in tumor progression and as a potential therapeutic target. Due to limited available findings on Th17 in mNSCLC cases, further research insights are required to decipher the upstream regulatory networks that govern Th17 cell differentiation and IL-17 secretion within the TME of NSCLC.

#### Tumor-associated neutrophils

Tumor-associated neutrophils (TANs) are infiltrating subsets of neutrophils found within tumors. Initially, TANs are recruited from the peripheral neutrophil pool by chemokines and subsequently transferred to the tumor stroma [561]. Similar to TAMs, TANs exhibit dual roles—either anti-tumoral (N1) or pro-tumoral (N2)—with functional polarization under the influence of adopted TIME [562–564].

Within the TME of NSCLC, TANs are among the most abundant immune cells [450] and contribute significantly to promoting NSCLC proliferation, invasion, and metastasis by synthesizing and secreting related proteases, including matrix metalloproteinases (MMPs) and neutrophil elastase (NE) [450]. For instance, TANs-secreted NE has been shown to promote tumor growth in a mouse model of lung ADC by degrading insulin receptor substrate-1 (IRS-1) [565]. Furthermore, NE mediates intercellular communication between TANs and NSCLC cells, enhancing tumor cell detachment and metastatic potential [566]. In another study, TANs showed high secretion and activity of matrix metalloproteinase-2 (MMP-2) and MMP-9 under the endogenous interferon-beta (IFN-β) inhibition within the TME, demonstrating an N2-type TAN phenotype that promotes lung cancer development [567].

TANs were also reported to induce EMT of lung ADC cells in vitro and enhance their migration activity [568, 569]. In a mouse model of lung cancer, TANs were shown to upregulate SNAI1 expression and enhance EMT in cancer cells, which further contributes to the advanced invasion and TAN infiltration into tumors [570]. Likewise, in vivo studies have demonstrated that TANs support the development of a tumor-supportive environment by inducing TNF-α and NO-dependent apoptosis of non-activated CD8^+^ T cells [571]. Besides, imaging mass cytometry and genomic analyses of paired samples have shown that metastasizing NSCLC clones exhibit significantly increased neutrophil infiltration compared to non-metastasizing tumors [572].

In conclusion, TANs can promote NSCLC progression and metastasis based on the TME. Given the limited research available on TAN functions in NSCLC metastasis, further studies are needed to elucidate the involved cellular and molecular pathways mediating the phenotype and functional plasticity of TANs in NSCLC patients.

## Outlook and challenges

NSCLC is a slow-progressing yet persistent and heterogeneous disease driven by a combination of somatic mutations and epigenetic dysregulation in key oncogene-associated signaling pathways. This heterogeneity allows NSCLC to acquire metastatic capabilities, making metastasis a major contributor to its high mortality rate. Unfortunately, the specific cellular communications and molecular pathways that enable the expansion of an immunosuppressive state during NSCLC metastasis—along with the mechanisms that allow tumor cells to detach from primary sites, evade immune surveillance, and establish colonization at distant metastatic sites—remain principally unclear. However, based on the available research, it can be estimated that specific NSCLC metastasis may be influenced by tumor genetic heterogeneity as well as the activity and population of immune cells in the TME. While these factors have been partially validated, significant progress could be achieved by mapping cellular heterogeneity, clonal expansion, and the dynamic functional alterations or communications among various cells within the TME that promote tumorigenesis. This might be accomplished through rapid analysis of spatial and single-cell transcriptomics data using advanced deep learning generative models.

### Artificial intelligence and omics integration in NSCLC management

Clinically, radiomics techniques are the standard approach for detecting lung cancer metastasis, modalities such as PET, CT, and MRI to predict tumor biology, cancer subtype, treatment response, and prognosis [573]. However, most NSCLC patients are diagnosed at an advanced stage or already present with metastases before the primary tumor is detected. Previous studies have shown that tumor cells can migrate to lymph nodes approximately 4.26 ± 0.74 years before the primary lung tumor is diagnosed [574]. For pleura metastases and distant metastases, dissemination occurs on average around 2.11 ± 0.33 years prior to primary tumor detection [574]. These findings align with a previous pan-cancer analysis—including breast, colorectal, and lung cancers—that estimated the metastatic seeding time for Lung cancer to be roughly 3.6 years before diagnosis [198]. Consequently, there is a critical need to identify biomarkers for lung cancer metastasis at the earliest stages, even when cancer cells remain in a pre-metastatic state without evident organ-specific tropism—a challenge not addressed by conventional diagnostic methods.

Advances in diagnostic techniques, such as immunohistochemical analysis using biomarkers like TTF-1, p63, p40, and cytokeratin 5/6, have significantly enhanced the accuracy of NSCLC subtyping. Additionally, substantial efforts have been made to integrate multi-omics approaches, including genomics, transcriptomics, and proteomics, for a deeper understanding of the molecular signatures unique to NSCLC subtypes. For instance, proteogenomic and histological analysis reveals that regardless of specific histologic classification, NSCLC subtypes exhibiting overexpression of the PI3K/Akt pathway are associated with a higher propensity for metastasis and poorer survival outcomes [575]. While preliminary histologic assessments, impression, and integrated multi-omics approaches are feasible in most cases, there remains a subset in which an accurate stage cannot be reliably determined during pathological evaluation [48]. For example, accurate classification of poorly differentiated and mixed NSCLC subtypes—and the distinction between true primary and metastatic tumors, particularly in the SCC subtype case [38]—can be especially challenging when relying solely on immunohistochemical staining and morphologic parameters.

Recent breakthroughs in artificial intelligence (AI)–based pathology have led to promising advances in lung cancer management, including quantitative analysis of histopathological features, prediction of biomarkers, and immunotherapy efficacy [576–580]. For instance, artificial intelligence (AI) models have been developed to distinguish ADC and SCC, as well as to differentiate lung cancer from healthy tissue [581–583]. Moreover, AI models have demonstrated superior accuracy in distinguishing benign from malignant lung nodules and in predicting histological subtypes [584, 585]. For example, combining radiomics with multimodal data and deep learning models has been shown to accurately predict *EGFR* mutation status in mNSCLC, which offers promising clinical value for early detection and personalized treatment [586–589]. Also, a combined model integrating clinical, radiomics, and deep learning features from PET/CT outperforms individual models in predicting LNM in NSCLC, and with Shapley additive explanations (SHAP)-based interpretability, it offers an accurate and practical tool for improving preoperative staging and reducing false-positive diagnoses [590]. In another study, a novel deep learning model, DeepGEM (an annotation-free AI method for predicting gene mutations), was developed using hematoxylin and eosin (H&E)-stained histopathological whole-slide images (WSIs) from primary lung cancer biopsies. This model has effectively linked histological phenotype with genetic mutation status*,* such as *EGFR and KRAS* mutations, and survival outcomes, including in LNM [591], thereby offering potential as an assistive diagnostic tool for mutation prediction and expediting treatment planning in lung cancer. Moreover, in contrast to traditional pathology assessment that provides diagnostic gold standards, AI-enhanced methods are now being employed to decode TME heterogeneity using H&E staining slides and IHC analysis [592–594]. For instance, AI–based pathology has been utilized to predict survival benefit from adjuvant therapy in early-stage NSCLC [595], as well as to identify and segment whole-slide images (WSIs) of lung tissue into tumor and non-tumor regions [582, 596]. Additionally, an AI-powered multimodal approach that integrates H&E staining and CT imaging has demonstrated complementary and synergistic effects in predicting clinical outcomes of ICI therapy in advanced NSCLC, including in the PD-L1 high-expression subgroup [597, 598]. Although some studies have reported errors, AI models have demonstrated superior detection capabilities compared to human experts in early diagnosis and screening [599–603]. Altogether, the integration of large histological and molecular diagnostic datasets with AI models enables highly sensitive and accurate analysis, significantly improving the identification of subtype-specific biomarkers and therapeutic targets.

Accumulating evidence in tumor biology indicates that the primary tumor's microenvironment may hold predictive value for future metastatic progression [166, 604]. Accordingly, disease stage remains the most widely used prognostic indicator in cancers, including NSCLC. However, although it offers general risk stratification for patient groups, it frequently lacks the precision to determine which individuals will ultimately develop metastatic disease. As a result, histopathologic evaluation—even when supplemented with molecular biomarkers—cannot reliably predict the metastatic potential of NSCLC, particularly in early-stage patients, where accurate risk assessment is critical for guiding treatment decisions [605]. These limitations highlight the necessity for integrating molecular profiling techniques to enhance diagnostic precision and staging accuracy in lung cancer. In this direction, transcriptomic profiling offers deeper biological insights and permits the classification of lung cancer into molecular subtypes based on the gene expression patterns associated with tumor growth and differentiation [98, 606].

Alongside the discovery of prognostic gene signatures, gene expression profiling has also revealed functional drivers of NSCLC metastasis. By comparing the expression profiles of highly metastatic cells with their weakly metastatic isogenic counterparts, researchers have efficiently identified candidate metastasis regulators, including both metastasis-promoting [607, 608] and metastasis-suppressing genes [609, 610]. In addition, genomic screens assessing gain and loss-of-function mutations, cross-species integrative genomic analyses, and computational reanalysis of large-scale genomic profiling datasets have significantly contributed to identifying clinically relevant functional mediators of NSCLC metastasis [611, 612]. However, it remains unclear whether the dysregulation of these key genes directly impacts the morphological appearance of the tumor tissues. Nevertheless, through the systematic analysis of transcriptomic-histopathology association, gene expression–linked morphological changes can be elucidated, offering deeper insight into tumor cell morphology at the molecular level [613]. In this context, a recent study reported that AI-based models are capable of classifying the transcriptomic subtypes of NSCLC using fully automated machine learning methods [614]. Supporting this, Cheng et al. [615] used RNA sequencing data from TCGA Lung cancer cohorts and applied a deep neural Network to predict metastasis status in NSCLC. Their approach revealed 31 transcripts via least absolute shrinkage and selection operator (LASSO) analysis, achieving the highest precision among the tested models [615]. Moreover, through the application of multiple machine learning algorithms focused on the diagnosis, prognosis, and immunotherapy in NSCLC, Wang et al. [616] revealed ARHGAP11A as a potential biomarker and therapeutic target linked to cancer stemness and LNM. Also, integration of AI-based models with multi-omics data, including proteomics and metabolomics, to predict metastasis and treatment outcomes in mNSCLC, as recently reviewed elsewhere [577]

Conclusively, AI emerges as a transformative tool in the clinical management of NSCLC, enabling the integration of radiomics, pathology, and multi-omics data to decode tumor-immune dynamics and predict therapeutic outcomes. However, one of the major obstacles to effective AI implementation is the lack of high-quality training data—often limited by high acquisition costs, stringent privacy regulations, and restricted data sharing, particularly about novel drug targets or treatments [617, 618]. Additionally, available datasets often suffer from missing information, technical variability, standard errors, biases, and inherent biological complexity, all of which undermine the reliability and generalizability of AI models. Model interpretability also remains a significant challenge, particularly in translating AI tools into clinical practice. Moving forward, the development of robust ethical frameworks, cross-institutional data validation, and clinical integration of AI-derived biomarkers will be essential for the advancement of AI-driven cancer management. Furthermore, effective AI model validation must extend beyond imaging and molecular features to incorporate key human- and disease-specific parameters such as genotype, physiological context, and cancer-specific TME heterogeneity. Accounting for these biological variables is essential for developing AI models that are not only accurate but also clinically meaningful and generalizable across diverse, cancer-specific patient populations.

### TNM staging and oligometastatic stratification

Recently, the IASLC published the 9^th^ edition of the pathological staging for lung cancer, which includes updates to the N2 and M1c subcategories. Although the 9^th^ edition demonstrated improved prognostic accuracy compared to the 8^th^ edition in a multicenter validation study that also included SCLC [619], a retrospective study reported challenges in preoperatively predicting the newly subdivided N2 stage [620]. In another study, Kim et al. [621] observed that the 9^th^ edition of the TNM classification lacks patient-specific clinical information, such as medical history and comorbidities. The same study also highlighted that variations in diagnostic and treatment modalities—stemming from institutional, national, and geographic differences—may potentially impact survival outcomes [621]; ^th^erefore, authors suggested a rigorous external validation of the 9^th^ TNM staging system using large, independent datasets from institutions with standardized protocols [621].

 The concept of oligometastatic disease has emerged as a distinct clinical state that lies between localized cancer and widespread systemic metastases. It represents an intermediate phase in the metastatic cascade, where the tumor has begun to spread but remains limited in number and location, potentially allowing for curative-intent treatment. Within this framework, further subclassifications have been proposed to capture the dynamic nature of metastatic disease evolution. For instance, oligoprogression refers to the progression of several metastatic lesions in an otherwise stable background of oligometastatic or even polymetastatic disease. In contrast, oligopersistent disease describes an induced state in which previously polymetastatic disease responds to therapy and is reduced to fewer than five active metastatic sites—a concept that has been reviewed in recent literature [622]. This evolving understanding of metastatic heterogeneity is mirrored in updates to the TNM classification system. Over successive editions, the M1 category has been progressively stratified—from two subcategories in the 7^th^ TNM edition, to three in the 8^th^, and four in the 9^th^—underscoring the significance and complexity of accurately defining heterogeneity and clinical relevance of metastatic burden [182, 623]. Despite these advancements, oligometastatic burden remains part of a biological and clinical continuum, without a definitive threshold distinguishing oligometastatic from polymetastatic disease states [624–626]. This lack of clear demarcation poses challenges for uniform staging and treatment planning. Currently, there is no international consensus on the definition of oligometastatic disease in the context of NSCLC [627]. However, the IASLC recommends a working definition of no more than five metastases across a maximum of three organs [628]. All organs, with the exception of diffuse serosal metastases and bone marrow involvement, are included within oligometastatic disease [628]. Additionally, the clinical relevance of defining oligometastatic NSCLC becomes especially significant when radical treatment is technically feasible and associated with an acceptable toxicity level [628].

Clinically, a commonly used criterion to distinguish oligometastatic from polymetastatic disease is the presence of up to five PET-defined distant metastases [629–631]. Despite its high diagnostic accuracy, PET imaging has limitations—particularly in polymetastatic cases—where reviewing and interpreting imaging reports can be challenging [632]. Even though PET scans are re-evaluated in cases when initial reports were inconclusive, counting lesions in polymetastasized patients remains ambiguous. Furthermore, small and/or non-avid metastases may go undetected, underscoring a key limitation of PET scans even in the context of their overall reliability as a cross-sectional imaging modality [633]. These insights emphasize that continued refinement of definitions and staging criteria for oligometastatic disease are essential for standardized clinical application. For instance, an optimal definition of oligometastatic disease should incorporate factors that predict disease biology and behavior, as patient outcomes are not solely determined by the number of metastatic lesions but also by their aggressiveness. This is illustrated by the fact, for instance, that patients with oligometastatic NSCLC presenting with a single metastasis generally have a better prognosis than those with multiple metastatic lesions [634]. Thus, additional research is needed to identify biological and clinical markers that can predict metastatic potential and treatment responses in specific patients or metastatic lesions [635].

Looking forward, future iterations of the TNM system may benefit from formally integrating molecular, radiomic, and immunologic biomarkers to better stratify metastatic disease beyond traditional anatomical criteria. Such a multidimensional approach could enable more personalized treatment strategies, enhance prognostic precision, and refine the selection criteria for local ablative therapies and systemic interventions in oligometastatic patients. Altogether, these insights emphasize the need for individualized assessment, ideally guided by prospective trials, histological and molecular profiling, and expert clinical judgment.

### Therapeutic challenges and rethinking immunotherapy

Clinical management of NSCLC has undergone a major transformation with the identification of actionable oncogenic drivers and the development of corresponding molecular-targeted therapies (TKIs), shifting treatment paradigms from a purely histology-based approach to molecular-targeted therapies [636–638]. Also, in non-oncogene-driven NSCLC, ICIs targeting PD-1 or PD-L1 have emerged as the cornerstone of first-line therapy or in combination with platinum-doublet chemotherapy (PT), regardless of histology or PD-L1 expression [269]. Despite the unceasingly rising number of targetable oncogenic alterations and associated therapies, real-world clinical experience reveals differential efficacy compared with that in clinical trials [639–642]. Together, these findings highlight a topic of paramount clinical relevance: identifying resistance mechanisms and developing new therapeutic strategies to overcome resistance, prolong survival, and maintain quality of life in this palliative setting.

In oncogene-addicted NSCLC, a key challenge of the precision medicine era is understanding and overcoming the development of resistance to targeted therapies, particularly those involving TKIs. In this direction, several intrinsic factors have been associated with therapeutic resistance in NSCLC [643, 644]. For instance, EMT activation through zinc finger e-box binding homeobox 1 (ZEB1) expression has been observed to mediate resistance against EGFR inhibitors in the *EGFR*-mutant NSCLC subtype [645]. From another perspective, *MET* amplification is a well-established mechanism of acquired resistance to EGFR inhibitors [646, 647]. Increasing recent evidence suggests that *MET* amplification consistently mediates acquired resistance across several other oncogene-driven NSCLC molecular subsets following TKI treatment. In response, combination strategies may help overcome the genomic heterogeneity underlying drug resistance; however, overlapping toxicities often necessitate dose adjustments, which can limit the effectiveness of these drug combinations [648]. Additionally, lineage transformation of tumor cells—such as transdifferentiation into neuroendocrine carcinoma, squamous cell carcinoma, or undergoing epithelial-mesenchymal transition (EMT)—has been identified as another mechanism contributing to resistance against molecularly targeted TKIs. This phenomenon is best described in the *EGFR*-mutant NSCLC subtype [4, 649], but has been progressively noticed in NSCLC with other driver mutations such as *ALK*, *KRAS,* and *RET* [650–652]. For instance, SCLC Transformation from NSCLC has been reported in 3%–14% of patients with *EGFR*-mutant NSCLC who gained resistance to first- and second-generation EGFR-TKIs [647, 653], and in 5%–20% of those with acquired resistance to the third-generation EGFR-TKI, osimertinib [646, 654], representing that SCLC transformation can happen regardless of the EGFR-TKIs generation. Although less frequent compared to EGFR-mutant NSCLC patients, SCLC transformation has also been reported in *ALK*-rearranged and *ROS1*-rearranged NSCLC following TKIs therapy [655, 656]. Additionally, SCC Transformation was estimated in 1.1%–14% of *EGFR*-mutant ADC patients with acquired resistance to EGFR-TKIs [657–659]. Similarly, histologic conversion from ADC to SCC has also been documented in patients resistant to *KRAS*^G12C^ inhibitors [660]. The common genetic changes and potential molecular mechanisms of gained therapeutic resistance attributable to histologic transformation of tumor cells in NSCLC have been reviewed elsewhere [661]. Although no promising therapeutic agents are yet designed for histologically transformed tumors, clinical trials for transformed SCLC are underway. All these findings together present a major clinical challenge, particularly due to the lack of robust resistance detection criteria and the limited post-osimertinib treatment options currently available [662].

The advancements of immunotherapies alone or in combination with chemotherapy have ushered in a new era in medicine, enabling improved management of advanced-stage NSCLC [275, 663–665]. However, the efficacy of ICIs is observed in patients with high PD-L1 expression and absence of oncogenic driver mutations such as *EGFR*, *ALK*, *ROS1*, *MET*, *KRAS*, or *STK11/LKB1* [666]. Supporting this, a retrospective study revealed that ICIs have limited activity in NSCLC patients with driver gene alterations [667]. Moreover, predictive biomarkers for ICIs responsiveness remain elusive, with only 20%–40% of patients benefiting from current therapies [11, 277, 668]. Also, phase III RCTs investigating beneficial therapeutic effects of new combination strategies have not reported substantial improvement for the overall population [289–292], although evidence suggests that certain patients may still derive substantial benefit [292]. As a result, up to 70%–85% of patients exhibit primary or acquired resistance to PD-1 blockade [669], even with combination therapy involving chemotherapy and/or anti-CTLA-4 agents [665]. Thus, the progress in effective immunotherapy strategies development for oncogene-addicted NSCLC is hindered by the lack of reliable predictive biomarkers for patient selection and limited knowledge of the optimal integration of ICIs with targeted therapies. In this context, it is hypothesized that biomarker-selected clinical trials may yield better outcomes than non-selected trials, such as TROPION-LUNG01 (NCT04656652) [670] and EVOKE-01 (NCT05089734) [671]. Nonetheless, it remains unclear whether ICIs—whether administered alone or in combination with chemotherapy or targeted agents—offer comparable benefits to patients harboring specific oncogenic driver mutations as observed in the broader, unselected NSCLC population.

Aside from PD-L1, the only other FDA-approved biomarker for solid tumors treated with pembrolizumab (as of 2020) is tumor mutational burden (TMB) [672]. TMB represents the number of somatic DNA variants per megabase (Mb), and a threshold of ≥ 10 mutations/Mb has been associated with favorable ICB outcomes in some studies [673, 674]. However, TMB’s reliability has been questioned, and it has not received approval in the European Union (EU). Reasons include the lack of standardized laboratory methods and variable cutoff levels (ranging from 10–20 mutations/Mb) proposed in different studies [673–676]. Contradictory findings have also been reported, with some patients showing clinical responses despite low TMB, and vice versa [675, 677, 678]. Additionally, the Society for Immunotherapy of Cancer (SITC) consensus and the panel in Schoenfeld et al. agree that pathological response extent and longitudinal biomarkers—such as circulating cell-free tumor DNA (cfDNA) and immunological markers—may serve as promising biomarkers, pending validation in prospective studies [679, 680]. In the context of cfDNA testing, liquid biopsy plays an important parallel role, especially when tumor access through surgical or radiological biopsy is limited [662]. However, liquid biopsies have limitations, including low nucleic acid concentrations (dependent on tumor burden), insufficient information on phenotypic switching, and low gene fusion detection rates [681]. Due to the absence of dependable biomarkers demonstrable by DNA sequencing of liquid biopsy samples, lineage transformation may remain undetected unless a repeat tissue biopsy is performed upon development of resistance.

To address resistance, the SITC has outlined three distinct scenarios of resistance to PD-1 pathway blockade: primary resistance, secondary resistance, and progression after treatment discontinuation [682]. A separate SITC criterion also addresses resistance to PD-1 pathway blockade in combination therapies involving ICIs and chemotherapy [680, 683]. According to these definitions, irrespective of monotherapy or combination therapy, primary resistance refers to progression within the first six months of ICI therapy, while secondary resistance occurs after at least six months of initial benefit [680, 683]. An expert panel focused on NSCLC has defined acquired resistance as disease progression following any objective response—excluding stable disease—regardless of the treatment duration [679]. However, there is ongoing debate about whether stable disease should be considered a clinical benefit prior to the onset of acquired resistance, as response evaluation criteria in solid tumors (RECIST) criteria for stable disease encompass a wide range of response patterns [679]. As understanding and overcoming the complex mechanisms of resistance after immunotherapy remains a significant clinical challenge, it is the focus of numerous ongoing clinical trials [684]. Additionally, a promising approach is integrating immunomodulatory therapies into sequential treatment guided by biomarkers, including combinational signatures identified through AI to improve outcomes and reduce tolerability, as explored in the European I3-Lung project [685].

In summary, the poor prognosis and outcome in mNSCLC can be attributed to four key factors: (i) tumor cell dispersal and survival in a "hostile" environment, including rare metastatic sites, which may promote highly aggressive tumor cell phenotypes; (ii) challenges in treating mNSCLC at unusual sites, especially when metastasis occurs in a single distant location; (iii) limited targeted therapy efficacy due to the emergence of new genomic alterations in a subset of cases; and (iv) inevitable resistance development following prolonged molecular therapy, often involving additional oncogenic driver mutations. Therefore, a comprehensive understanding of the pathophysiological and molecular networks in mNSCLC is essential. Gaining deeper insight into cellular and immune-related mechanisms will be crucial for the development of more effective therapeutic strategies. In this perspective, Genomic profiling plays a crucial role in guiding first-line treatment decisions for patients with advanced NSCLC. Obtaining a comprehensive molecular profile of the tumor before starting treatment is essential for several reasons: (1) to accurately identify and initiate the most effective therapy, given the superior outcomes observed with first-line tyrosine kinase inhibitors (TKIs) compared to chemotherapy in patients with oncogene-driven tumors [686, 687]; (2) to prevent inappropriate treatment, since frontline immunotherapy—whether alone or combined with chemotherapy—is not recommended for these patients [688]; and (3) to avoid TKI-related toxicities that may arise if TKIs are administered following immunotherapy-based treatments [689–691].

### Immune dynamics and challenges in NSCLC

With the rise of single-cell sequencing and spatial omics, the comprehensive participation of TME in the initiation, progression, and development of lung cancer has been recognized [305, 692]. However, the clinical management of NSCLC remains challenging due to our limited understanding of the interplay between oncogenic mutations, EMT, CSCs, and immune cell dynamics (such as MDSCs, TILs, and TAMs) within the tumor immune environment (TIME), which collectively drive immune evasion, tumor progression, and metastasis of NSCLC. In addition, non-transformed immune and stromal cells, along with their dynamic crosstalk with cancer cells, have been implicated in regulating early tumor development and playing key roles in tumor progression, including advanced disease stages and metastasis [604]. Among the immune cells in the TME, TAMs and MDSCs are especially noteworthy for their roles in tumor development and metastasis. For example, TAMs and myeloid MDSCs are known to produce immunosuppressive molecules such as arginase I, interleukin (IL)−10, or transforming growth factor beta (TGF-β) [693, 694]. Additionally, Tregs, which express CD25 and FoxP3, contribute to immune suppression by depleting IL-2 from the local environment, thereby hindering immune activation. Tregs also express inhibitory molecules such as cytotoxic T lymphocyte antigen 4 (CTLA-4), which interacts with CD80/CD86 on antigen-presenting cells (APCs) to block co-stimulatory signaling [695, 696]. Similarly, tumor-infiltrating lymphocytes (TILs), particularly αβ T cells (CD4^+^ and CD8^+^) [697], and their subpopulations are also reported for their considerable functional roles in TME. For instance, a higher density of CD3^+^, CD4^+^, CD8^+^, and CD20^+^ TILs is suggestive of an antitumoral effect, while FOXP3^+^ regulatory T cells are associated with immunosuppression and poor prognosis in NSCLC [698].

Notably, immune cell infiltration in the tumor is also dependent on the TNM stage [450, 451]; thus, stage-dependent profiling of immune cells in the NSCLC tumors may provide insights into disease development. Tumor cells release various immunosuppressive molecules—such as TGF-β, IL-6, prostaglandin E2, chemokine ligand 2, and colony-stimulating factor 1—which can suppress CD8^+^ T cell function and facilitate the recruitment of immunosuppressive cells, reviewed elsewhere [696]. Also, lung cancer cells have been shown to reduce the surface expression of MHC class I and tumor antigens (human leukocyte antigen-G (HLA-G), thereby enabling immune evasion [699]. Both tumor cell-associated and soluble HLA-G have been reported in the blood of NSCLC patients, and are associated with advanced TNM stages, suppressed NK-cell-mediated cytolysis, and reduced OS [700, 701].

High PD-L1 expression is also frequently observed in NSCLC tumor cells [702], and is strongly associated with suppressed maturation of tumor-infiltrating DCs and reduced T cell infiltration [703]. Furthermore, recent studies have reported that CD8⁺ PD-L1⁺ double-positive T cells correlate with increased tumor burden, contributing to a 'hot' yet immunosuppressive TME [704]. Importantly, due to the highly heterogeneous nature of NSCLC, genomic and epigenetic alterations also contribute to immune exhaustion or depletion of TILs. For example, *KRAS* and *LKB1/STK11* mutations in NSCLC subtypes are associated with impaired T cell infiltration [705, 706], while EMT has been particularly shown to regulate the PD-L1 expression via epigenetic reprogramming, allowing NSCLC cells to escape immune surveillance [316]. Inactivation of the *STK11* gene has been reported to weaken innate immune response by suppressing the stimulator of interferon genes (STING) pathway through epigenetic mechanisms [707]. These resistant tumors typically display a distant immune contexture characterized by low cytotoxic immune cell infiltration and high levels of MDSCs [708]. Given the heterogeneity of NSCLC, patient-specific profiling of immune cell infiltration, mutation profiles, and epigenetic modifications within the TME is essential for developing effective, personalized therapies aimed at enhancing T cell activation and persistence.

Moreover, the major subtypes of NSCLC, SCC and ADC, have been shown to exhibit distinct transcriptomic profiles and TMEs [709–711]. For instance, a higher number of TANs has been reported in SCC than in ADC, whereas macrophages are more abundant in ADC subtype [450, 712]. In genetically engineered mouse models (GEMMs), SCC models such as *Lkb1*^*fl/fl*^* (*SL*); Pten*^*fl/fl*^ (LP) and *Sox2*^*LSL/LSL*^* Pten*^*fl/fl*^*; Cdkn2ab*^*fl/fl*^ (Sox2PC) were found to have higher TAN levels but fewer macrophages by comparison to ADC GEMMs [713, 714]. Additionally, *EGFR*- and *KRAS*-driven ADC mouse tumors exhibit distinct lymphocyte populations, suggesting that oncogenic drivers can shape the TIME [715]. The absence of LKB1 in NSCLC has also been linked to reduced neutrophil and T-cell infiltration in the TME [716]. These findings have led to the hypothesis that squamous histological classification (i.e., histotype) rather than genetic alterations (i.e., genotype), primarily determines the immune contexture [717].

Given the conditions, understanding the complex crosstalk among immune cells, cancer cells, and the TME across disease stages—and elucidating the molecular mechanisms that promote tumor progression—may yield new prognostic markers and enable the development of more effective combination therapies aimed at restoring anti-tumor immunity. In this direction, integrating deep learning models with clinical data—including pre-treatment PET/CT scans, TNM staging, and tumor localization—alongside immune profiling (e.g., TAM and TAN interactions, co-stimulatory signaling dynamics) within the NSCLC microenvironment, may substantially improve prognostication and risk identification in diagnosed cancer patients.

### Limitations of humanized mouse models in NSCLC research

Despite much attention given to the development of new therapies for cancer, many patients either do not respond or experience disease relapses after an initial positive response. The foremost challenge is the lack of suitable human specimens for research, and secondly, there is a limited understanding of the specific therapy resistance mechanisms that emerge during antineoplastic treatment. These limitations underscore the need for models that can accurately replicate efficacy and resistance (both primary and acquired) to the selected treatment(s). In this direction, recent reviews have emphasized the strengths and weaknesses of existing metastasis models [718–721].

Over the past decades, considerable efforts have been devoted to developing preclinical cancer models, including cell line-derived xenografts (CDX), patient-derived xenografts (PDX), genetically engineered mouse models (GEMM), and NOD scid gamma mice (NSG), to advance understanding of mNSCLC biology and pathogenesis [722–725]. However, available evidence showed that ex vivo models often exhibit limitations to completely replicate the physiological complexity of the in vivo system [725–728]. Similarly, GEMMs, which incorporate clinically relevant genetic alterations identified in lung cancer patients—such as *KRAS*, *p53*, and *ALK* mutations—develop tumors orthotopically, yet often exhibit longer latency periods and sporadic metastases [729–731], which limits their experimental feasibility. Additionally, GEMMs typically exhibit a lower mutational burden compared to human tumors, which further compromises their ability to model immune responses and accurately recapitulate the complexity of the human TME [721, 732]. As a result, an evolving area of investigation in cancer immuno-oncology (IO) is the use of preclinical humanized mouse models—immunodeficient mice co-engrafted with both human tumors and components of the human immune system—which allow for the study of human-specific immune responses [733, 734]; a summary of humanized mice studied in mNSCLC is given in Table 11. For instance, humanized immunodeficient mice such as NSG (NOD/SCID/IL2Rγ^null^) can exhibit a functionally reconstituted surrogate human immune system [735]. Yao et al. [736] demonstrated that treatment with ex vivo-expanded double-negative T cells (DNTs) induced tumor regression in a NSCLC CDX model using humanized NSG mice. In another study, Meraz et al. [737] reported that *NPRL2* gene-therapy stimulated antitumor activity in *KRAS/STK11* mutant/aPD-1–resistant tumors via DC-mediated antigen-presentation and activation of cytotoxic immune-cells in humanized NSG mice.

**Table 11:** Humanized mouse models in metastatic NSCLC therapeutic studies

Tumor Type	Mouse Strain (genotype)	Humanized approach	Therapy strategy	Ref
*KRAS/STK11*-mutant metastatic NSCLC (A549 cells)	NSG (NOD.Cg-*Prkdc*^*scid*^*Il2rg*^*tm1Wjl*^/SzJ)	Human CD34⁺ HSCs from cord blood injected into irradiated NSG mice	*NPRL2* gene therapy ± anti-PD-1 (pembrolizumab)	[737]
*EGFR*-mutant, *MET*-amplified metastatic NSCLC	NSG (NOD.Cg-*Prkdc*^*scid*^*Il2rg*^*tm1Wjl*^/SzJ)	PBMC-engrafted humanized model	Pemetrexed + CD73 inhibition	[750]
*KRAS/LKB1* (*STK11*)–mutant metastatic NSCLC (A549-luc cells)	NSG (NOD.Cg-*Prkdc*^*scid*^*Il2rg*^*tm1Wjl*^/SzJ)	CD34^+^ + HSC transplantation (cord blood)	Combined therapy: systemic tumor suppressor gene (TUSC2/NPRL2) nanovesicles + chemotherapy (carboplatin) + anti–PD-1 (pembrolizumab)	[751]
Human NSCLC (AXL^+^, A549, and NSCLC PDX, subcutaneous and lung metastasis models)	HuNOG-EXL (NOD.Cg- Prkdc^scid^I*l2rg*^null^ IL-3/GM-CSF-transgenic)	CD34^+^ + HSC engraftment in NOG-EXL mice (humanized NOG-EXL)	CAR T cells targeting AXL, combined with local microwave ablation	[752]
Human NSCLC cell line Xenografts (EGFR-mutant and wild-type lines, PDL1^high/low^)	NSG (NOD.Cg-Prkdc^*scid*^Il2rg^tm1Wjl^/SzJ)	Adoptive transfer of Activated human T cells Engineered with a PD-L1^−^ specific CAR	CAR T-cell immunotherapy (anti–PD-L1 CAR, ± low-dose radiotherapy)	[753]
Metastatic NSCLC (CDX)	NSG (NOD.Cg-*Prkdc*^*scid*^*Il2rg*^*tm1Wjl*^/SzJ)	Human CD34⁺ HSCs → reconstituted immune system	Ex vivo expanded DNT (double-negative T cell) therapy	[736]
Human NSCLC xenografts (cell-line and PDX; subcutaneous and metastatic models)	NSG (NOD.Cg-*Prkdc*^*scid*^*Il2rg*^*tm1Wjl*^/SzJ)	Fresh cord blood CD34^+^ HSC transplant (4–8 week engraftment)	Anti-PD-1 therapy (pembrolizumab, nivolumab)	[754]
Human NSCLC PDX (patient derived Tumor xenografts)	NSG (NOD.*Cg-Prkdc*^*scid*^*IL2rg*^*tm1Wjl*^/SzJ with > 25% hCD45^+^	Cord blood CD34^+^ HSC Transplantation (cord-blood engraftment)	Anti-PD-1 immune Checkpoint blockade (pembrolizumab)	[755]
Human NSCLC xenografts (EGFR, e.g H460)	NSG (NOD.Cg-Prkdc^scid^Il2rg^tm1Wjl^)	Human T cells transduced ex vivo with EGFR specific CAR (adoptive T-cell transfer)	CAR T-cell immunotherapy (anti-EGFR CAR T cells)	[756]

Despite substantial progress, humanized mouse models still present notable limitations. For instance, mouse models of cancer often failed to accurately recapitulate fundamental aspects of tumor biology, TME, and therapeutic responses—including resistance mechanisms—when compared to clinical outcomes. One illustrative case is the use of *NPRL2* gene-therapy, which demonstrated promising antitumor efficacy in reversing anti-PD-1 resistance in *KRAS/STK11*-mutant NSCLC using an NSG mouse model. Although this model offers advantages over traditional murine systems for immunotherapy research, it still lacks the full complexity and diversity of the human immune system and TME [737–739]. Supporting this, NSG mice are not specifically HLA-matched to the human CD34^+^ hematopoietic stem cells (HSCs) used for immune reconstitution, which affects immune compatibility and tumor-immune interactions [740, 741]. Most studies have also been limited to a small number of tumor models to replicate only certain pathological features of human disease and lack complex genetic and phenotypic heterogeneity in clinical NSCLC [706, 742]. Moreover, humanized mice often exhibit incomplete reconstitution of myeloid lineages and limited cytokine cross-reactivity, both of which may impair immune cell-tumor interactions and affect therapeutic responses [743].

In transplantation studies, the growth behavior of human xenografts can vary significantly within the murine host, suggesting that in vivo experiments may be more reflective of experimental conditions rather than the intrinsic behavior of human tumor cells. Also, the short time frames used to evaluate tumor response in these models limit conclusions about long-term efficacy, durability of response, and mechanisms of immune escape. Available studies suggest that lung CSCs contribute to therapy resistance via several mechanisms, including asymmetric cell division, slow division kinetics or quiescence, and the expression of drug efflux pumps and DNA repair proteins [744–746]. However, many of these findings are derived from murine models, and their clinical relevance to human tumors remains uncertain. For instance, the commonly used murine stem cell antigen-1 (Sca-1) has no human homolog [747]. Furthermore, lifespan differences between mice and humans can also influence stem cell proliferation dynamics. As a result, modeling tumor-immune cell interactions is challenging and poses limitations for therapy development.

Nonetheless, despite ongoing variability in outcomes and persistent uncertainties regarding the translational relevance of preclinical animal models to human clinical settings, a growing body of evidence supports the utility of humanized mouse models as valuable tools for investigating the role of immune cell populations in tumor control and for assessing the efficacy of immunotherapies [748, 749]. Simultaneously, methodologies for humanization are continually advancing, enabling more accurate preclinical assessment of in vivo therapeutic responses. While animal models remain indispensable for evaluating the efficacy of new treatments, their inherent limitations necessitate cautious interpretation of findings and underscore the need for subsequent validation in more representative and clinically relevant model systems.

## Conclusion

Given the complexity and clinical challenges associated with NSCLC diagnosis and treatment, future research must prioritize unravelling the immune profiles of organ-specific metastases. Additionally, identifying novel molecular drivers and biomarkers will be crucial for advancing general and/or personalized therapies. In this direction, emerging technologies such as CRISPR, spatial single-cell sequencing, and integrative multi-omics hold an immense promise for elucidating intratumoral heterogeneity and the plasticity of cancer and immune cells. Moreover, an innate understanding of EMT dynamics and immune evasion during metastasis could also provide insights into the development of new combinatorial therapeutic strategies targeting both cancer cells and immunosuppressive TME. For instance, new immunotherapy strategies might include targeting tumor-associated immune cells, for example, to enhance the infiltration of functional T cells or regenerate their anti-tumor function, in combination with therapy to inhibit the pro-tumorigenic functions of specific cells in the TME. Considering the complexity of the mechanisms governing the infiltration, activation, and survival of various tumor-associated immune cells within the TME of NSCLC subtypes, and the fact that these mechanisms may differ between patients due to physiology and/or pathological state, further investigation is needed to understand the regulatory roles of tumor-associated immune cells in the TME. Additionally, identifying reliable biomarkers or assays for monitoring immune responses in NSCLC requires further exploration. With accumulating clinical experience and advancing scientific research, collaborative efforts among pathologists, oncologists, and researchers are essential to refining classification standards, enhancing diagnostic accuracy, and ultimately improving clinical outcomes and personalized care strategies for NSCLC patients.

## Review criteria

Information for this Review was compiled by searching the Google Scholar, MEDLINE and PubMed databases using the following search terms: “non-small-cell lung cancer”, “NSCLC histology”, “genetics of NSCLC”, “Clinical data and NSCLC metastasis”, “Clinical trials and metastatic NSCLC”, “Humanized mouse and metastatic NSCLC”, “immune cells and NSCLC metastasis”, “Cancer stem cells and NSCLC metastasis”, “Artificial intelligence and NSCLC”, “EMT and NSCLC metastasis”. References from relevant articles were checked for additional Material. Only English language articles published between 2015 and 2025 were included during the literature search from the respective databases.

## Data Availability

No datasets were generated or analysed during the current study.
